# A multi-analytical approach to studying the chemical composition of typical carbon sink samples

**DOI:** 10.1038/s41598-023-35180-x

**Published:** 2023-05-17

**Authors:** Maria Luisa Astolfi, Lorenzo Massimi, Mattia Rapa, Rita Rosa Plà, Raquel Clara Jasan, Mabel Beatriz Tudino, Silvia Canepari, Marcelo Enrique Conti

**Affiliations:** 1 Department of Chemistry, Sapienza University of Rome, P.le Aldo Moro 5, 00185 Rome, Italy; 2 CIABC, Sapienza University of Rome, P.le Aldo Moro 5, 00185 Rome, Italy; 3 Department of Environmental Biology, Sapienza University of Rome, P.le Aldo Moro 5, 00185 Rome, Italy; 4 C.N.R. Institute of Atmospheric Pollution Research, Via Salaria, Km 29,300, Monterotondo St., 00015 Rome, Italy; 5 Department of Management, Sapienza University of Rome, Via del Castro Laurenziano 9, 00161 Rome, Italy; 6 Departamento Química Nuclear, Gerencia Química Nuclear y Ciencias de la Salud (GAATN), Centro Atómico Ezeiza, Comisión Nacional de Energía Atómica (CNEA), Av. Presbítero J. González y Aragón 15 (CP B1802AYA), Ezeiza, Buenos Aires, Argentina; 7 INQUIMAE, Departamento de Química Inorgánica, Analítica y Química Física, Facultad de Ciencias Exactas y Naturales, Universidad de Buenos Aires, Buenos Aires, Argentina

**Keywords:** Infrared spectroscopy, Metals, Environmental sciences, Chemistry, Analytical chemistry

## Abstract

Peatlands in southern South America (Tierra del Fuego region, TdF) play a key role in the ecological dynamics of Patagonia. It is, therefore, necessary to increase our knowledge and awareness of their scientific and ecological value to ensure their conservation. This study aimed to assess the differences in the distribution and accumulation of elements in peat deposits and *Sphagnum* moss from the TdF. Chemical and morphological characterization of the samples was carried out using various analytical techniques, and total levels of 53 elements were determined. Furthermore, a chemometric differentiation based on the elemental content of peat and moss samples was performed. Some elements (Cs, Hf, K, Li, Mn, Na, Pb, Rb, Si, Sn, Ti and Zn) showed significantly higher contents in moss samples than in peat samples. In contrast, only Mo, S and Zr were significantly higher in peat samples than in moss samples. The results obtained highlight the ability of moss to accumulate elements and to act as a means to facilitate the entry of elements into peat samples. The valuable data obtained in this multi-methodological baseline survey can be used for more effective conservation of biodiversity and preservation of the ecosystem services of the TdF.

## Introduction

Peatlands, composed almost entirely of decomposing plant material, represent the world's major land-based carbon sink^1^ contributing to the resilience increase of ecosystems^2^. However, they occupy only 3% of the global land area^3–5^. Peatlands’ fundamental function of regulating the carbon cycle contributes to climate change mitigation^6,7^. They also play a key role in the conservation of biodiversity by ensuring habitat for various living species, paleoenvironmental archives, and archaeological remains and play a special role in hydrological cycle regulation through water storage, groundwater recharge, and drought and flood mitigation^7,8^. In addition, peat and *Sphagnum* moss are recognized globally as valuable economic resources for their use as fuel and horticultural substrate, respectively^9–11^. However, peat is a non-renewable resource^6^. In recent years, new strategies for the wise use of peatlands and new management policies with limited mining concessions have been proposed^6,12^.

In temperate South America, peat bogs are dominated by *Sphagnum* moss^13^ and are poor in nutrients (ombrotrophic)^14^. Ombrotrophic bogs are hydraulically isolated and receive all nutrients, including major and trace elements, by atmospheric depositions and precipitation^15^. Several studies have shown that ombrotrophic peat bogs are also useful archives for Hg deposition records^16,17^. Accumulation rates and concentrations of Hg in ombrotrophic peatlands are influenced by peat humification processes in addition to site location and anthropogenic and natural sources^16^. In fact, the ability of peat to bind metals is determined by the high content of humic substances and the developed surface^18,19^. Carboxyl and phenolic functional groups present in humic substances, which make up peat organic matter, influence peat chemical properties such as metal complexation, buffering capacity, acid–base reactions, and cation exchange capacity^20,21^. Also, *Sphagnum* moss, due to the high cation exchange capacity of its surface, is suitable for monitoring atmospheric element depositions^11,22–25^. However, only some elements were investigated, while others could be bound by *Sphagnum* moss, as pointed out by other authors^24^. In addition, the elemental composition of deposited dust is necessary to understand the dust's geochemical cycle and its relationship to climate change^26^.

Limited and fragmentary information is available on Patagonian peatlands^27^; new studies must be undertaken to improve knowledge of these areas and evaluate the possible contamination by chemicals in the future. A chemical-physical characterization as complete as possible could also allow the creation of artificial products to avoid the depletion of natural resources and protect biodiversity. For this reason, our main objectives are to determine and compare the total contents of 53 elements (Al, As, B, Ba, Be, Bi, C, Ca, Cd, Ce, Co, Cr, Cs, Cu, Eu, Fe, Ga, H, Hf, Hg, K, La, Li, Lu, Mg, Mn, Mo, N, Na, Nb, Ni, O, P, Pb, Rb, S, Sb, Sc, Se, Si, Sm, Sn, Sr, Te, Th, Ti, Tl, U, V, W, Yb, Zn, and Zr) in peat and living *Sphagnum* moss from eight sites in the Tierra del Fuego (TdF, south Patagonia) using several analytical techniques and chemometric tools [Principal Component Analysis (PCA) and stepwise variable selection]. Other objectives are to study the types and amounts of functional groups by Fourier Transform Infrared (FTIR) spectroscopy to highlight possible correlations between the elements’ accumulation and the chemical structures of peat and *Sphagnum* moss.

## Material and methods

### Study area

**Figure 1:**
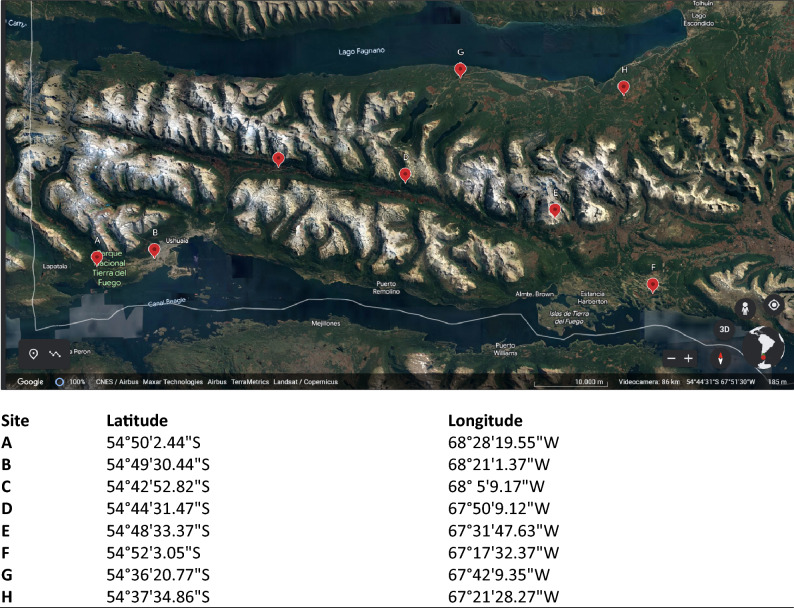
Map of the study area in Argentina, South America. Locations of the sampling sites are marked in the detailed map of the north area of the Tierra del Fuego region (South Patagonia). Datum for geographical coordinates is based on the World Geodetic System 1984 (WGS84) ellipsoid. Data map: Google, CNES/Airbus, Maxar Technologies, Airbus, TerraMetrics, Landsat/Copernicus.

Tierra del Fuego is located at the southern end of the American continent (Fig. 1). It extends southeast of the Magellan Strait between the Atlantic and Pacific oceans. The archipelago consists of the main island, the Big Island of TdF, often called simply TdF or Big Island, with an area of 48,100 km^2^ and a myriad of smaller islands. The main island is divided politically between two nations: 38.6% belongs to Argentina (east), while 61.4% belongs to Chile (west). The biggest cities on the main island in the Argentine part are the Rio Grande and Ushuaia, with 57,000 people.

The climate of TdF is influenced by latitude, the nearby presence of the Antarctic ice mass, ocean currents, and the nature of the land itself. The nearby Antarctic continent exposes the archipelago to the presence of cold air masses, especially in winter, while the presence on three sides of ocean waters simultaneously tends to keep the temperature range between summer and winter low. The region has an oceanic climate with short cool summers and long wet, moderately cold winters. The main bog concentration in TdF is located in the east, where the precipitation is about 700–900 mm year^−1^ ^6^. These peatlands are linked to the climate and soil and water chemical properties^28^. Peatlands are the dominant ecosystem in the Argentine part of the TdF, and their coverage reaches 90% in some eastern watersheds^28^.

### Sampling

Eight ombrotrophic and mostly undisturbed peat bogs and *Sphagnum* moss samples were collected in 2018. Peatland patches were sampled in duplicate and selected according to the peatland map of Roig et al.^29^. A map of the location of eight sampling sites (A, B, C, D, E, F, G, and H) and the geographical coordinates of the selected points are shown in Fig. 1. Except for site B, which is located in Ushuaia, the most populated city of the archipelago, and near the International Airport, the other sites are in low anthropogenic impacted areas. Peat samples were collected using a field drill, and cores were extracted to a depth of 20 cm, divided into 10 cm segments, and filled in plastic bags. The peat and *Sphagnum* moss samples were dried at 40 °C for two days, ground (using a ceramic mortar and pestle), and homogenized in the laboratory. All samples were stored at 4 °C until analysis.

### Analysis of powder samples

#### Carbon, H, N, S and O analysis

An elemental analyzer (EA 1110 CHNS/O, CE Instruments, United Kingdom) was used to determine the percentages of C, H, N, O, and S in all samples (~ 5 mg)^30^. The analytical procedure consists of the combustion of the sample (1000 °C) followed by catalytic oxidation and reduction processes, separation of the gases produced in the gas-chromatographic column, and analysis of the same with a thermal conductivity detector (set at 290 °C); signal processing and determination of the percentage of elements present in the sample with the EAGER 200 program.

#### High resolution morphology study and semi-quantitative analysis

Scanning electron microscope (SEM) analysis was performed with a high-resolution field emission SEM (HR-FESEM; model Auriga 405; Carl Zeiss Microscopy GmbH, Jena, Germany) equipped with an energy dispersive spectrometer for X-ray microanalysis (XEDS, model Quantax; Bruker, Berlin, Germany). Before analysis, a small portion of the powder sample was fixed to specific support and coated with a thin layer of C by a sputtering machine (Q150T Turbo-Pumped Sputter Coater/Carbon Coater; Quorum Technologies Ltd., East Sussex, United Kingdom) to make its surface conductive. HR-FESEM XEDS acquisitions were performed under a high vacuum (10^−6^ hPa) at 20 keV accelerating voltage. Micrographs were acquired by secondary electron detector (SED) at a working distance (WD), magnification, spot size, and tilt angle conditions properly adjusted to optimize image resolution. The microanalysis was performed at WD and magnification ranging from 3.0 to 7.8 mm and 100× to 45,000×, respectively.

#### Mercury analysis

Mercury was determined using an advanced mercury analyzer (AMA-254, Altec Ltd., Prague, Czech Republic) according to EPA method 7473^31^. Each sample (~ 20 mg) was analyzed in duplicate, and a certified reference material (BCR 482) was used for the analytical procedure’s quality assurance and control. Trueness bias percentage and precision were 2.3 and 3.2%, respectively. The limit of determination and quantification (LOD and LOQ, respectively), defined as the Hg concentration corresponding to three and ten times the standard deviation of the blanks (n = 10), was 0.0001 and 0.004 mg kg^−1^, respectively. A Hg reference standard solution (1,002 ± 7 mg L^−1^ in 10% HNO_3_; SCP Science, Baie D’Urfé, Quebec, Canada) in 1% v/v HNO_3_ (67% suprapure, Carlo Erba Reagents, Milan, Italy) was used to prepare calibration solutions (in the range of 0–50 μg L^−1^). All aqueous solutions were prepared using deionized water with a resistivity of 18.2 MΩ cm^−1^, generated by an Arioso Power I RO-UP Scholar UV deionizer (Human Corporation, Songpa-Ku, Seoul, Korea). More details on the AMA method are presented in previous work^32^.

#### Element analysis by INAA

Instrumental neutron activation analysis (INAA) was performed at the Nuclear Analytical Techniques Laboratory (Ezeiza Atomic Centre, Argentine National Atomic Energy Commission) for the determination of Eu, Hf, Lu, Sc, Sm, Th, and Yb mass fractions.

Peat and *Sphagnum* moss samples, about 25 and 300 mg, respectively, were irradiated at the RA-3 reactor (thermal flux 3.1013 cm^−2^ s^−1^, 8 Mw) for 4.5 h. Two measurements with 7 and 30-day-decay counted from the end of irradiation, were done using GeHP detectors (30% efficiency, 1.8 keV resolution for the 1332.5 keV 60Co peak). The Gamma Vision software was employed to acquire the gamma spectra, and elemental mass fractions were calculated using software developed at the NAA laboratory. For quality control purposes, Andesite ACH-1 and Grass (Poeaceae) from WEPAL 2011–4 were used as control samples for peat and *Sphagnum* moss analysis, respectively^33^. The following certified reference materials were used for the calibration of the INAA: SRM Coal Fly Ash 1633c (National Institute of Standards and Technology -NIST, Gaitherburg, MD, USA), CRM Soil GBW 07,405 (GSS-5) (Institute of Geophysical and Geochemical Exploration, Langfang, China) and RM IAEA Lichen 336 (International Atomic Energy Agency, Seibersdorf Laboratories, Seibersdorf, Austria). The average results (n = 3) obtained from the analysis of the certified reference materials by INAA showed a good agreement with the certified data (Table S1). The INAA LODs are shown in Tables 1 and S2.

**Table 1:** Results of the total elemental content (mg/kg d.w.) in peat and *Sphagnum* moss by AMA, ICP-MS, ICP-OES, and INAA (n = 16 for each matrix).

Element	Instrument	Wavelength^a^ or isotope^b^	LOD	LOQ	Peat						*Sphagnum* moss						
					n > LOD (%)	Mean	SD	Median	Min	Max	n > LOD (%)	Mean	SD	Median	Min	Max	*p*-value^c^
Al	ICP-OES	237.312^a^	5	20	100	1100	620	910	409	2200	100	2510	2560	1340	264	7790	ns
As	ICP-MS	75^b^	0.2	0.8	81	2.1	4.1	0.5	< 0.2	13.5	85	1.2	1.0	0.9	< 0.2	2.9	ns
B	ICP-MS	11b	5	20	6	< 5	–	< 5	< 5	9	0	< 5	–	< 5	< 5	< 5	–
Ba	ICP-OES	233.527^a^	0.5	2	100	32	26	23	9	95	100	40	26	27	3	82	ns
Be	ICP-MS	9^b^	0.02	0.07	75	0.054	0.052	0.032	< 0.02	0.179	82	0.066	0.061	0.043	0.010	0.182	ns
Bi	ICP-MS	209^b^	0.01	0.03	62	< 0.01	–	0.008	< 0.01	0.016	62	0.022	0.020	0.014	< 0.01	0.067	ns
Ca	ICP-OES	317.933^a^	30	100	100	2080	1210	1750	703	3940	100	2590	1500	2370	1020	6200	ns
Cd	ICP-MS	112^b^	0.008	0.03	100	0.074	0.043	0.057	0.034	0.167	100	0.130	0.082	0.131	0.026	0.285	ns
Ce	ICP-MS	140^b^	0.009	0.03	100	3.2	3.7	1.4	0.4	10.9	100	5.9	5.8	4.8	0.2	20.4	ns
Co	ICP-OES	228.615^a^	0.4	1	98	1.7	3.1	0.5	< 0.4	9.9	98	1.4	1.1	1.4	< 0.4	3.3	ns
Cr	ICP-OES	267.716^a^	0.6	2	94	3.2	2.0	2.8	< 0.6	9.4	80	3.7	4.0	1.6	< 0.6	12.1	ns
Cs	ICP-MS	133^b^	0.002	0.008	100	0.047	0.025	**0.044**	0.013	0.099	100	0.23	0.19	**0.18**	0.01	0.61	**
Cu	ICP-MS	65^b^	0.2	0.7	100	2.6	1.7	1.9	0.8	6.2	100	3.6	3.0	2.6	0.6	9.4	ns
Eu	INAA	-	0.06	–	25	0.09	0.12	< 0.06	< 0.06	0.38	63	0.14	0.15	0.11	< 0.06	0.52	–
Fe	ICP-MS	56^b^	1	5	100	2680	4150	997	379	13200	100	3320	2730	2940	123	8010	ns
Ga	ICP-MS	71^b^	0.003	0.009	100	0.30	0.13	0.28	0.11	0.52	100	0.86	0.83	0.55	0.05	2.78	ns
Hf	INAA	–	0.2	–	100	3.9	3.8	**2.4**	0.5	16.2	100	17.5	19.5	**11.6**	2.6	79.8	***
Hg	AMA	–	0.0001	0.004	100	0.044	0.021	0.034	0.019	0.076	100	0.063	0.032	0.055	0.022	0.116	ns
K	ICP-OES	766.491^a^	20	60	100	221	102	**198**	94	417	100	1740	938	**1380**	687	3570	***
La	ICP-MS	139^b^	0.005	0.02	100	1.3	1.5	0.6	0.2	4.1	100	2.3	2.4	1.6	0.1	8.5	ns
Li	ICP-MS	7^b^	0.02	0.06	100	0.126	0.076	**0.136**	0.021	0.255	100	1.2	1.3	**0.6**	0.0	3.9	**
Lu	INAA	-	0.01	–	44	0.03	0.04	< 0.01	< 0.01	0.12	69	0.06	0.05	0.04	< 0.01	0.18	ns
Mg	ICP-MS	24^b^	2	7	100	957	334	953	498	1420	100	1320	609	1320	433	2470	ns
Mn	ICP-OES	257.610^a^	0.3	1	100	109	244	**23**	4	738	100	212	135	**196**	11	448	**
Mo	ICP-MS	98^b^	0.1	0.3	85	0.22	0.20	**0.14**	< 0.1	0.65	31	0.1	0.1	** < 0.1**	< 0.1	0.3	**
Na	ICP-OES	589.592^a^	6	20	100	413	164	**401**	166	696	100	1220	991	**761**	482	3680	***
Nb	ICP-MS	93^b^	0.005	0.02	100	0.158	0.083	0.142	0.044	0.311	100	0.22	0.15	0.17	0.01	0.43	ns
Ni	ICP-MS	60^b^	0.09	0.3	100	1.7	2.3	0.8	0.3	7.8	94	2.3	1.9	2.1	0.1	5.3	ns
P	ICP-OES	185.878^a^	10	30	100	252	166	224	61	619	100	324	157	331	41	639	ns
Pb	ICP-MS	208^b^	0.03	0.09	100	0.71	0.28	**0.64**	0.22	1.28	100	3.8	3.6	**2.3**	0.1	10.3	**
Rb	ICP-MS	85^b^	0.02	0.05	100	0.74	0.46	**0.61**	0.26	1.75	100	3.5	2.7	**2.6**	0.9	9.9	***
S	ICP-OES	181.972^a^	60	200	100	2190	673	**2190**	1220	3320	100	722	580	**504**	239	2100	***
Sb	ICP-MS	121^b^	0.2	0.6	0	< 0.2	–	< 0.2	< 0.2	< 0.2	56	< 0.2	–	< 0.2	< 0.2	< 0.2	–
Sc	INAA	–	0.01	–	100	0.67	0.44	0.57	0.14	1.50	100	1.64	1.68	1.08	0.09	5.52	ns
Se	ICP-MS	76^b^	0.5	2	50	0.5	0.3	0.4	< 0.3	1.0	19	< 0.5	–	< 0.5	< 0.5	1.2	–
Si	ICP-OES	288.158^a^	300	850	100	3830	2250	**3200**	1080	8520	100	23800	30100	**12000**	781	100000	***
Sm	INAA	–	0.01	–	100	0.42	0.55	0.15	0.05	1.75	100	0.68	0.72	0.47	0.04	2.46	ns
Sn	ICP-MS	118^b^	0.02	0.06	85	0.051	0.030	**0.045**	< 0.01	0.114	85	0.15	0.13	**0.13**	< 0.02	0.45	**
Sr	ICP-MS	88^b^	0.2	0.6	100	30	15	23	14	54	100	26	12	26	10	56	ns
Te	ICP-MS	125^b^	0.02	0.08	0	< 0.01	–	< 0.01	< 0.01	< 0.01	0	< 0.01	–	< 0.01	< 0.01	< 0.01	–
Ti	ICP-OES	334.941^a^	1	3	100	78	47	**74**	22	168	100	302	322	**187**	13	1060	**
Th	INAA	–	0.05	–	88	0.49	0.51	0.27	0.03	1.73	94	1.02	0.92	0.80	< 0.05	3.00	ns
Tl	ICP-MS	205^b^	0.001	0.004	100	0.014	0.011	0.008	0.004	0.033	100	0.041	0.031	0.042	0.002	0.088	ns
U	ICP-MS	238^b^	0.003	0.009	100	0.073	0.050	0.061	0.015	0.165	100	0.106	0.097	0.090	0.005	0.334	ns
V	ICP-MS	51^b^	0.09	0.3	100	2.3	1.3	2.2	0.6	4.5	100	5.8	5.8	4.1	0.3	20.6	ns
W	ICP-MS	182^b^	0.1	0.4	0	< 0.1	–	< 0.1	< 0.1	< 0.1	0	< 0.1	–	< 0.1	< 0.1	< 0.1	–
Yb	INAA	–	0.1	–	38	0.2	0.3	< 0.1	< 0.1	0.9	69	0.3	0.3	0.2	< 0.1	1.1	–
Zn	ICP-MS	66^b^	1	4	44	2.2	2.5	** < 1**	< 1	7.4	100	19	10	**18**	8	40	***
Zr	ICP-OES	339.198^a^	1	5	100	7370	7200	**4580**	798	30100	100	675	712	**440**	108	2930	***

^a^Wavelength selected for the elemental analysis by ICP-OES.

^b^Isotope selected for the elemental analysis by ICP-MS.

^c^Non-parametric Mann Whitney test was applied: “– “ = not determined; “ns” = not significant at *p* > 0.05; “*” = *p* < 0.05; “**” = *p* < 0.01; “***” = *p* < 0.001. Numbers in bold in the same row indicate significant differences.

#### FTIR

A Fourier transform infrared spectroscopy (FTIR; IR Affinity Miracle 10; Shimadzu Scientific Instruments, Columbia, MD, USA) was used to provide information about functional groups' types and relative abundance. The IR spectra were recorded in the 4,000–600 cm^−1^ with a resolution of 5.0 cm^−1^.

### Analysis of digested samples

The total contents of 41 elements (Al, As, B, Ba, Be, Bi, Ca, Cd, Ce, Co, Cr, Cs, Cu, Fe, Ga, K, La, Li, Mg, Mn, Mo, Na, Nb, Ni, P, Pb, Rb, S, Sb, Se, Si, Sn, Sr, Te, Ti, Tl, U, V, W, Zn, and Zr) were measured by ICP-OES (Vista MPX CCD Simultaneous; Varian, Victoria, Mulgrave, Australia) and ICP-MS (820-MS; Bruker, Bremen, Germany) equipped with a collision–reaction interface (CRI). Arsenic, Cr, Fe, Mn, Se, and V were analyzed using ICP-MS with the CRI mode and H_2_ and He (99.9995% purity; SOL Spa, Monza, Italy) as cell gases; the other elements by ICP-MS in standard mode with the exception of Al, Ba, Ca, Cr, K, Na, S, Si, Ti and Zr, which were determined by ICP-OES. The ICP-MS and ICP-OES optimized instrumental parameters are summarized in a previous study^34^. Multi-element standard solutions (VWR International, Milan, Italy) were used for instrumental calibration curves (seven-point). Yttrium at 0.005 and 0.2 mg L^−1^ (Panreac Química, Barcelona, Spain) for ICP-MS and ICP-OES, respectively, and Sc, Rh, In, and Th at 10 mg L^−1^ (Merck, Darmstadt, Germany) for ICP-MS only were used as internal standards.

Total digestion of the powder samples was performed in a close microwave oven system (Ethos1 Touch Control; Milestone, Sorisole, Bergamo, Italy) using an acid mixture of HCl–HF–HNO_3_, according to Bettinelli et al.^35^, Astolfi et al.^36^, and Gaeta et al.^37^. Briefly, weighed amounts (~ 100 mg) of the samples were transferred into polytetrafluoroethylene vessels; to these, 1 mL H_2_O_2_ (30% superpure, Merck, Darmstadt, Germany), 1 mL HCl (30% suprapure, Carlo Erba Reagents, Milan, Italy) and 3 mL HNO_3_ (method M1) or 1 mL HF (40% superpure, Sigma-Aldrich Chemie GmbH, Steinheim, Germany), 1 mL HCl and 3 mL HNO_3_ (method M2) were added. The solutions obtained were heated with microwave energy for 40 min using a program with temperature ramps up to 180 °C. The obtained digests were diluted to 20 mL with deionized water and filtered (0.45 μm cellulose nitrate membrane; GVS Filter Technology, Indianapolis, IN, USA). The samples by method M1 were further diluted 1:10 with deionized water. After these procedures, the digests obtained with M1 or M2 method were analyzed by ICP-MS or ICP-OES, respectively. Method blanks were periodically analyzed alongside the samples to check for any losses or cross-contamination. The LODs and LOQs are shown in Table 1. A standard reference material (NIST 1515, Gaithersburg, MD, USA) was analyzed for quality control purposes. For all the certified elements (Al, B, Ba, Ca, Cd, Ce, Co, Cr, Cu, Fe, K, La, Mg, Mn, Mo, Na, Ni, P, Pb. Rb, S, Sb, Sr, U, V, W, and Zn), the trueness bias percentage was between -9.7 (La) and 17% (Ca) of the expected value, and the precision as repeatability was between 0.5 (K)-25% (Sb)^36^. For the other elements (As, Be, Bi, Cs, Ga, Li, Nb, Se, Si, Sn, Te, Ti, Tl, and Zr) there are indicative levels in the peat and *Sphagnum* moss samples. For the elements in common between the different analytical techniques (As, Ce, Co, Cr, Cs, Fe, La, Rb, Sb, and Zn), a quality control of the data was carried out through intertechnical comparisons.

### Statistical analysis

Statistical analyses were conducted using IBM® SPSS® Statistics 27 software (IBM Corp., Armonk, NY, USA). For each element, values below the LOD were replaced with a value equal to half the LOD^38,39^. When the percentage of values < LOD exceeded 30%, the element was excluded from the statistical dataset.

The differences in the sample concentration were tested by Kruskal–Wallis and pairwise post-hoc tests and Mann–Whitney test. Probability values from multiple pairwise comparisons were adjusted using Bonferroni corrections^40^.

Principal component analysis and variable selection by stepwise approach were performed with JMP 16 Pro (SAS Institute) to highlight possible sample grouping. Autoscaling pretreatment was applied on data matrices before the chemometric analyses.

## Results and discussion

### Morphological semi-quantitative surface characterization of peat and *Sphagnum* moss samples

**Figure 2:**
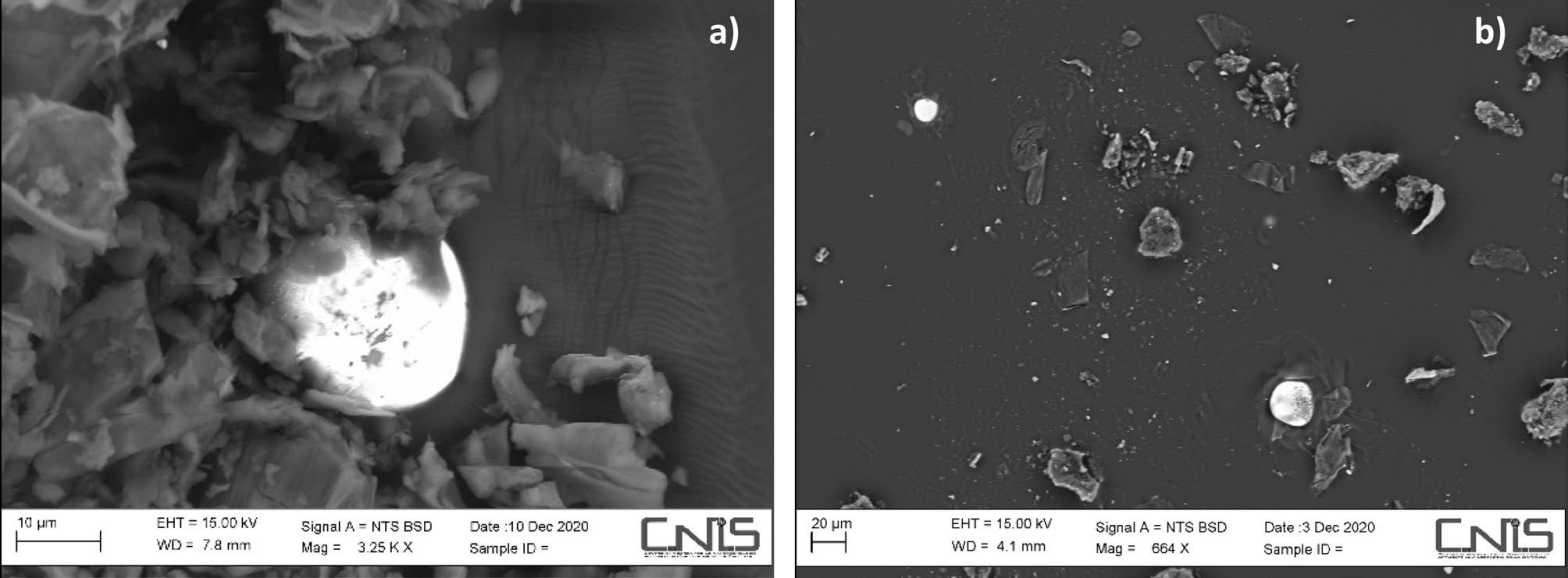
HR-FESEM micrographs evidencing the presence of spheroidal metal particles in (**a**) peat and (**b**) *Sphagnum* moss samples.

The HR-FESEM XEDS investigation showed morphology and surface composition of the peat and *Sphagnum* moss samples (Figs. 2 and S1–S4, and Tables S3 and S4). In all the samples the following elements Al, Ca, Cl, Mg, Na, O, P, S, and Si are present in variable percentages, while Cr and Cu are found only in peat and Fe, K, and N in *Sphagnum* moss (Tables S3 and S4). Oxigen (from 25.8 to 45.6% in peat and from 29.7 to 44.5% in *Sphagnum* moss) is the most abundant element. The surface of the peat samples appears layered and porous, while the *Sphagnum* moss samples show globular and fibrous formations. Backscattered electron images (Fig. 2) provide a differentiation between the organic matter (weak brightness) and the inorganic fraction (strong brightness) in the samples. In both matrices, there are metallic spherical particles (Fig. 2). The inorganic spheroidal particles present in the upper layers of peat are of predominantly anthropogenic origin such as industrial activities and coal combustion, since cosmic spherules (generally produced by combustion processes) are extremely rare in peat^41,42^.

### Carbon, H, N, O and S contents

The analysis of C, H, N, O and S contents confirmed the HR-FESEM XEDS results: O (from 40.8% in peat to 63.3% in *Sphagnum* moss) and C (from 31.8% in *Sphagnum* moss to 51.0% in peat) together with H (from 4.46 to 7.13% in *Sphagnum* moss) and N (from 0.29% in *Sphagnum* moss to 1.96 in peat) are the elements that have higher contents in both the matrices considered (Table 2).

**Table 2:** Elemental percentage contents in peat and *Sphagnum* moss samples by the EA 1110 CHNS/O analysis (n = 16).

Sample	Site	% C	% N	% H	% S	% O^a^	C/N	H/C	O/C
Peat	A	50.8	0.82	6.87	0.03	41.5	61.7	0.14	0.82
	B	50.8	1.17	7.12	0.11	40.8	43.6	0.14	0.80
	C	47.8	1.29	6.32	0.18	44.4	37.1	0.13	0.93
	D	48.8	0.75	6.69	0.01	43.8	65.1	0.14	0.90
	E	49.6	1.96	6.78	0.04	41.6	25.4	0.14	0.84
	F	51.0	1.17	6.50	0.04	41.3	43.7	0.13	0.81
	G	50.6	0.82	7.07	0.10	41.4	61.6	0.14	0.82
	H	49.1	0.73	6.78	0.04	43.3	67.8	0.14	0.88
	All	49.8	1.09	6.77	0.07	42.3	45.8	0.14	0.85
*Sphagnum* moss	A	31.8	0.43	4.46	0.06	63.3	74.8	0.14	1.99
	B	43.1	0.36	6.45	0.01	50.0	119	0.15	1.16
	C	39.3	0.57	5.77	0.03	54.4	68.8	0.15	1.38
	D	44.2	1.18	6.01	0.26	48.4	37.4	0.14	1.10
	E	45.1	0.63	6.76	0.08	47.4	72.1	0.15	1.05
	F	43.1	0.29	6.57	0.03	50.0	149	0.15	1.16
	G	49.1	1.67	7.13	0.18	41.9	29.4	0.15	0.85
	H	43.6	0.29	6.24	0.04	49.8	153	0.14	1.14
	All	42.4	0.68	6.17	0.09	50.7	62.7	0.15	1.19

^a^Determined by difference (excess result).

Peat is made up of organic material that contains about 50% C (Table 2), so C concentrations reflect changes in peat humification and related mass losses in peatlands^16^. The decomposition of organic material in peat leads to the formation of phenolic structures derived from lignin^43^. These structures are more difficult to degrade than proteins and sugars^43,44^. The O/C and H/C ratios can give information on the carbohydrate content and the percentage saturation of C within the organic molecule. Therefore, a higher aromaticity in the samples will be given by lower H/C ratios^43^; while a lower O/C ratio indicates a lower carbohydrate level and/or higher organic content in the peat sample^45^. The C/N ratios are also related to the decomposition processes of organic matter since the microbial consumption of organic substances determines a decrease in the abundance of C compared to N^16,46^. However, decomposition by microorganisms is limited due to the lack of nutrients in the peat and the low pH^16^. Therefore, the C/N ratio is used as an indicator of the degree of decomposition of peat and the mass loss of peat^16,46^. The C/N ratios in peat are an indicator of the degree of humification, where low C/N ratios indicate high humification of the peat^46^. Previously reported ratios of C/N values for peat and bog vegetation mainly ranged from 40 to 100^16,46^. Overall, the results obtained in this study (Table 2) are in agreement with those of the literature^16,43,46^. The samples with the highest C/N ratio are those in site H while those with the highest degree of humification are present in sites E for peat and G for *Sphagnum* moss. Generally, changes in the humification index are related to changes in environmental conditions, i.e. different climates and resulting differences in peat accumulation and decomposition^46^. The high C/N ratio, between 60 and 153 (sites A, D, G, and H for peat and A, B, C, E, F, and H for *Sphagnum* moss) suggests rapid peat growth under conditions of humidity while lower values may indicate drier marshes and, therefore, exposure of peat to aerobic decay for more extended periods^46,47^. Differences in vegetation can be explained by lower rainfall and higher nutrient input through marine sprays^48,49^.

The S content of peat consists of organic S, sulphate and sulphides^50^. Not much information is found in the literature on S in peat and *Sphagnum* moss. Organic S in Scottish peat is predominant with about 64% of total S being C-bonded sulphur and 27% ester sulphate S^51^. Some authors did not find a clear correlation between S intake and its concentration in peat^52^. The S levels measured in the *Sphagnum* mosses sampled in the Tierra del Fuego region range between 0.01 and 0.26%, which is similar to those reported for non-polluted sites^53^.

### Spectroscopic characterization of organic matter by FTIR

Figures S5 and S6 show the infrared spectra of the different samples, while Table S5 presents a summary of the main bands observed in peat and the assignments of the related functional groups. The spectra present wide bands typical of natural organic matter and are due to the superposition of single absorption bands^43^. The main features of the spectra are: a broad band around 3400 cm^−1^ due to O–H stretching of various groups like alcohol and phenols; two peaks at 2920 and 2850 cm^-1^ due to C–H stretching and characteristics of aliphatics (fats, wax, lipids); a region between 1720 and 1420 cm^−1^ assigned to C=O stretch of carbonyl and carboxyl groups (carboxylic acids and aromatic esters), aromatic C=C and asymmetric COO− group vibrations (lignin and other aromatics and aromatic or aliphatic carboxylates), and OH deformations and C=O stretch of phenols or C–H deformation (phenolic and aliphatic structures); and, finally, absorption bands in the 1100–1000 cm^−1^ region were allocated to combination of C–O stretching and O–H deformation of polysaccharides^46,54–56^.

### Chemical composition of peat and *Sphagnum* moss samples

For all elements analyzed in peat and *Sphagnum* moss samples, higher total levels of each element were found in *Sphagnum* moss than in peat samples except Mo, S and Zr (Table 1). The most abundant elements in both matrices were Al, Ca, Fe, S, Si, and Zr, ranging from 440 (Zr) to 12,000 (Si) mg kg^-1^. Cesium, Hf, K, Li, Mn, Na, Pb, Rb, Si, Sn, Ti, and Zn levels in *Sphagnum* moss were about 2 (Na) to 18 (Zn) times significantly higher than that in peat. In contrast, significantly higher levels of elements were found in the peat samples than in the *Sphagnum* moss samples for Mo, S, and Zr. Considering the element levels for each matrix and site (Tables S6 and S7), no significant differences were highlighted, showing the uniform distribution of elements in each matrix in the area under study.

Some elements, such as Pb and Hg, tend to bind strongly to organic material^50^. Lead is considered immobile and well-preserved in peat profiles^57,58^. Even most of the elements that represent the deposition of dust, such as Si and Zr, or others, such as Al, Ti, Sc and rare earth elements (REE), which are mainly identified as lithogenic tracers, should be immobile and stable^50^. However, some elements, such as Pb, tend to form organic complexes at low pH values and for this reason can move along peat profiles^50^.

Some studies showed that the increase in Hg concentration was linked to a greater decomposition of the peat (low C/N ratio) and not necessarily to a greater contribution from atmospheric depositions^59^. Our data, in agreement with other studies^60,61^, showed no correlation between Hg and peat decomposition (*p* < 0.05). This highlights a homogeneity in the composition of the sampled peat.

Our results match those of Wang et al.^25^, which show that moss accumulates most elements by deposition, including toxic elements, and can be a means of facilitating the accumulation of metal(loid)s in the soil. Furthermore, wet or dry atmospheric deposition of particles on the moss surface can subsequently be solubilized or washed away with precipitation^62–64^. Thus, peat can be enriched with metals and significantly contribute to elemental levels in moss^25^.

Each country has defined risk levels associated with different metal(loid) concentrations^65–67^. For example, Table S8 shows the threshold levels established by Legislative Decree 152/2006 and subsequent amendments and additions^68^ and the standards established in the Finnish legislation for contaminated soil^69^. Concerning Finnish legislation, the highest concentration levels are defined by the main land uses, i.e., industrial or transport sites and other land uses. The second level of concentration is the so-called “guide value”. If this is exceeded, the area presents a level of contamination with ecological risk (e) or health risks (t). Different guide values are set for industry and transport areas (highest guide value), and for all other land uses (lowest guide value). For the assessment of agricultural land, the threshold of lower guide values for the sample applies. The elemental levels in peat were all below regulatory standards, except As in site E (13.0 ± 1.0 mg/kg), which exceeds the Finnish threshold value of 5 mg kg^−1^. However, even if anthropogenic arsenic pollution is widespread, As in soil is generally considered mainly of geological origin, with a higher background concentration in clayey soils^66^.

### Chemometric analysis

The above presented results showed several differences between peat samples from moss samples. Chemometric analyses have been applied to confirm this differentiation and to visualize sample differences.

Usually, a chemometric treatment is needed when the sample size is large and exceed the variables’ number. Nevertheless, principal component analysis should be applied to reduced observations to perform an explorative analysis and to provide a graphical representation of a natural grouping of samples.

In this regard, t, a stepwise variables selection was carried out for all the analyses performed aiming to reduce the number of variables and select the most informative ones. The minimum Bayesian information criterion (BIC) was applied to point out which variables exhibited the best separation among peat and moss samples.

**Figure 3:**
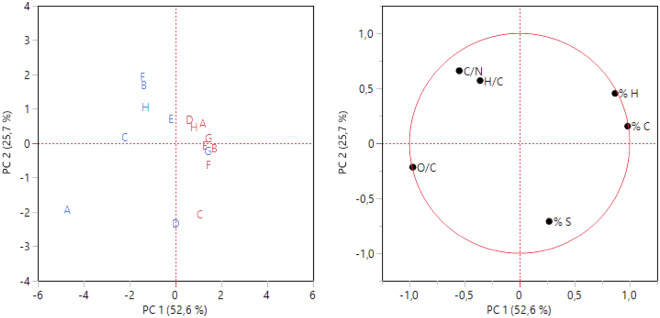
Scores and loading plots of PCA on selected variables for C, H, N, O and S contents. Red: peat, blue: moss.

The variables’ stepwise results for C, H, N, O and S contents were reported in Table S9; the only variables excluded were %N and %O. These findings match the analytical results differences. Principal component analysis was performed with the selected variables and reported in Fig. 3. The first two principal components accounted for 78.3% of the total variability. Peat samples (red) are all located in the right part of the plot, while the moss ones are in the left one, except the moss of the G site. The grouping of peat and moss samples is more evident on PC1, which was mostly influenced by %C and O/C variables. So, these variables seem to better characterize the samples’ grouping.

**Figure 4:**
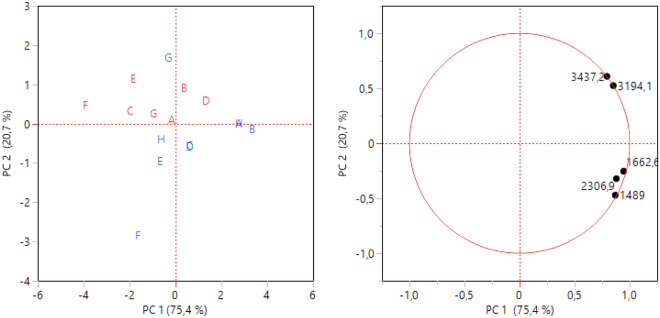
Scores and loading plots of PCA on selected variables for infrared spectra. Red: peat, blue: moss.

Regarding the infrared spectroscopic characterization, the variables selected by the stepwise approach were the wavelengths: 1489, 1662, 3194, 2307 and 3437 cm^−1^ (Table S10). It is noteworthy that these wavelengths fall in the typical range of lignin and cellulose compounds, as reported in Table S5. Therefore, the peat and moss samples’ differences in the organic matter should be attributed to these compounds. Principal component analysis plots for FTIR vibration bands were reported in Fig. 4. The first two PCs accounted for 96.1% of the total variance. Variables showed a similar contribution on PC1 and PC2. Moss samples (blue), except for the G site, are located in the lower part of the graph, while peat ones (red) are in the higher one.

**Figure 5:**
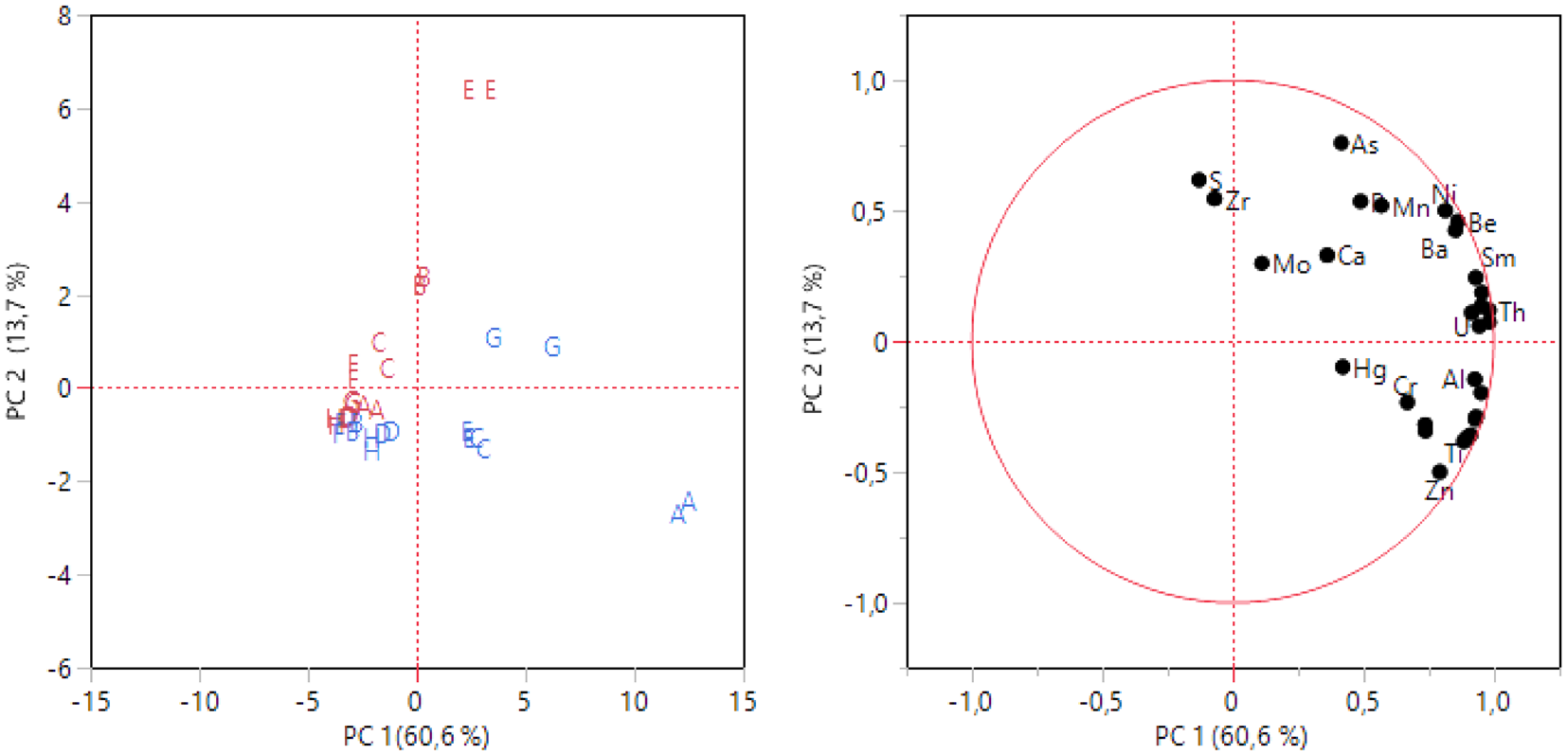
Scores and loading plots of PCA on selected variables for multi-elemental analysis. Red: peat, blue: moss.

In the multi-element analysis, the variable stepwise included twenty-nine of forty-nine elements determined, i.e. S, Ca, Hg, Mo, P, Zr, As, Hf, Be, La, U, Al, Ce, Ba, Mn, Ni, Sm, Th, Pb, Cr, Si, Lu, Ti, Sn, Cu, Rb, Ga, Zn and Bi (Table S11). Notably, the variables included to differentiate samples belong to different classes such as macro-elements, trace-elements, and REEs. This finding confirms the use of different element classes to characterize environmental samples. Principal component analysis has been performed with the selected elements (Fig. 5). The first two PCs explained 74.3% of the total variability with a partial grouping of peat and moss samples. In fact, moss samples from A, C, E and G sites are clearly located in the right part of the score plot, while the samples from the other sites appear partially overlapped with peat ones. The major differences between peat and moss are mainly due to the different Zn and As contents, while the similarities are attributable to the S and Zr contents.

## Conclusions

The elemental distribution and physicochemical characteristics in peat deposits and *Sphagnum* moss of the Tierra del Fuego region (south Patagonia) were estimated. Our study demonstrated the need to monitor the content of elements in the peatlands of Southern Argentina to assess the risk of anthropogenic pollution and moss's role in the metals' biogeochemical cycle. The anthropic impact could, in fact, endanger the ecosystems of peat bogs, sites of great ecological importance since they can be a refuge for rare flora and fauna species and, for this reason, play a fundamental role in the conservation of biodiversity and safeguard of ecosystem services. Therefore, it is important to design peat bog research and management strategies to increase the knowledge and awareness of scientific communities and local populations on the importance of these ecosystems. This study provides baseline information useful for the evaluation of the resilience capability of the Patagonian ecosystem in a mid-long term.

## Supplementary Information

Supplementary Information.

## Data Availability

The datasets generated during and/or analyzed during the current study are available from the corresponding author on reasonable request.
