# Supplementary material for: A multi-analytical approach to studying the chemical composition of typical carbon sink samples
Source: Sci Rep. 2023 May 17;13:7971. doi: 10.1038/s41598-023-35180-x (PMC10192350; doi:10.1038/s41598-023-35180-x)
Supplement: Supplementary file 1 — Supplementary Information. [file 41598_2023_35180_MOESM1_ESM.docx]

Supplementary material

**A multi-analytical approach to studying the chemical composition of typical carbon sink samples**

Maria Luisa Astolfi^1,2,^*, Lorenzo Massimi^3,4^, Mattia Rapa^5^, Rita Rosa Plà^6^, Raquel Clara Jasan^6^, Mabel Tudino^7^, Silvia Canepari^3,4^, Marcelo Enrique Conti^5^

^1^ Department of Chemistry, Sapienza University of Rome, P.le Aldo Moro 5, 00185, Rome, Italy

^2^ CIABC, Sapienza University of Rome, P.le Aldo Moro 5, 00185 Rome, Italy

^3^ Department of Environmental Biology, Sapienza University of Rome, P.le Aldo Moro 5, Rome, 00185, Italy

^4^ C.N.R. Institute of Atmospheric Pollution Research, Via Salaria, Km 29,300, Monterotondo St., Rome, 00015, Italy

^5^ Department of Management, Sapienza University of Rome, Via del Castro Laurenziano 9, 00161 Rome, Italy

^6^ Departamento Química Nuclear, Gerencia Química Nuclear y Ciencias de la Salud (GAATN), Centro Atómico Ezeiza, Comisión Nacional de Energía Atómica (CNEA), Av. Presbítero J. González y Aragón 15 (CP B1802AYA), Ezeiza, Buenos Aires, Argentina

^7^ INQUIMAE, Departamento de Química Inorgánica, Analítica y Química Física, Facultad de Ciencias Exactas y Naturales, Universidad de Buenos Aires, Buenos Aires, Argentina

*Corresponding author: marialuisa.astolfi@uniroma1.it

**Table S1.** Data obtained from the analysis of certified materials using INAA (n=3).

| **Element** | **Certified value** | | **Experimental value** | |  |
| --- | --- | --- | --- | --- | --- |
|  | **concentration** | **uncertainty/ confidence interval** | **concentration** | **uncertainty** | **Certified reference material** |
|  | **mg kg^-1^** | | **mg kg^-1^** | |  |
| **As** | 412 | 16 | 430 | 7 | Soil^a^ |
| **Br** | 24.2 | 1.6 | 24.1 | 1.6 | Grass^b^ |
| **Ca** | 5,453 | 251 | 5,200 | 800 | Grass^b^ |
| **Ce** | 68.4 | 6.6 | 63.5 | 4.8 | Andesite^b^ |
| **Co** | 18.8 | 1.1 | 19.6 | 0.9 | Andesite^b^ |
| **Cr** | 198 | 5 | 212 | 10 | Coal^c^ |
| **Cs** | 1.35 | 0.12 | 1.3 | 0.2 | Andesite^b^ |
| **Eu** | 1.3 | 0.2 | 1.2 | 0.06 | Andesite^b^ |
| **Fe** | 37,546 | 1,645 | 37,000 | 800 | Andesite^b^ |
| **Hf** | 6.80 | - | 6.99 | 0.80 | Coal^c^ |
| **K** | 25,870 | 1,330 | 25,000 | 5,400 | Grass^b^ |
| **La** | 34.8 | 2.3 | 34.1 | 0.6 | Andesite^b^ |
| **Lu** | 0.42 | 0.05 | 0.432 | 0.042 | Soil^a^ |
| **Na** | 3,293 | 227 | 3,270 | 220 | Grass^b^ |
| **Rb** | 117 | 6 | 118 | 14 | Soil^a^ |
| **Sb** | 0.049 | 0.042 | 0.047 | 0.009 | Grass^b^ |
| **Sc** | 12.9 | 0.4 | 12.6 | 0.1 | Andesite^b^ |
| **Sm** | 4.86 | 0.61 | 4.83 | 0.50 | Andesite^b^ |
| **Tb** | 0.014 | 0.012 - 0.016 | 0.0141 | 0.0075 | Lichen^d^ |
| **Th** | 6.81 | 0.48 | 6.55 | 0.17 | Andesite^b^ |
| **Yb** | 2.8 | 0.4 | 2.51 | 0.38 | Soil^a^ |
| **Zn** | 210 | - | 259 | 8 | Coal^c^ |

^a^ Soil, GBW 07405

^b^ Andesite ACH-1 and Grass (Poaceae) from Wepal 2011-4

^c^ Coal Fly Ash (NIST-1633b)

^d^ RM IAEA Lichen 336

**Table S2.** Results [mean and standard deviation (SD); mg kg^-1^ d.w.] from INAA of the elements also analyzed by ICP-MS and ICP-OES (n=16).

|  |  |  | **As** | **Ce** | **Co** | **Cr** | **Cs** | **Fe** | **La** | **Rb** | **Sb** | **Zn** |
| --- | --- | --- | --- | --- | --- | --- | --- | --- | --- | --- | --- | --- |
| **Sample** | **Site** | **LOD^a^** | 1 | 0.5 | 0.2 | 0.5 | 0.1 | 200 | 0.2 | 2 | 0.03 | 2 |
| **P** | **A** | Mean | <1 | 1.94 | 0.51 | 1.81 | <0.1 | 851 | 1.01 | <2 | <0.03 | 6.5 |
|  |  | SD | - | 0.54 | 0.01 | 0.53 | - | 18 | 0.20 | - | - | 1.2 |
|  | **B** | Mean | <1 | 8.8 | 1.36 | <0.5 | <0.1 | 3,620 | 4.35 | <2 | <0.03 | 3.1 |
|  |  | SD | - | 2.3 | 0.37 | - | - | 190 | 0.89 | - | - | 3.0 |
|  | **C** | Mean | <1 | 3.16 | 0.76 | 1.43 | <0.1 | 1,230 | 1.03 | <2 | <0.03 | 3.5 |
|  |  | SD | - | 0.40 | 0.01 | 1.66 | - | 200 | 0.18 | - | - | 3.5 |
|  | **D** | Mean | <1 | 1.0 | 0.69 | <0.5 | <0.1 | 1,460 | 0.73 | <2 | <0.03 | <2 |
|  |  | SD | - | 1.1 | 0.15 | - | - | 95 | 0.03 | - | - | - |
|  | **E** | Mean | 16.7 | 13.26 | 12.3 | <0.5 | <0.1 | 16,900 | 4.73 | <2 | <0.03 | <2 |
|  |  | SD | 1.3 | 0.64 | 0.5 | - | - | 620 | 0.42 | - | - | - |
|  | **F** | Mean | <1 | 0.51 | 0.50 | <0.5 | <0.1 | 792 | 0.29 | <2 | <0.03 | 3.1 |
|  |  | SD | - | 0.36 | 0.04 | - | - | 77 | 0.03 | - | - | 0.9 |
|  | **G** | Mean | <1 | 1.25 | 0.47 | 1.73 | <0.1 | 832 | 0.58 | <2 | <0.03 | 3.9 |
|  |  | SD | - | 0.02 | 0.01 | 0.31 | - | 25 | 0.05 | - | - | 0.5 |
|  | **H** | Mean | <1 | 0.64 | 0.36 | 0.51 | <0.1 | 427 | 0.30 | <2 | <0.03 | 3.3 |
|  |  | SD | - | 0.19 | 0.06 | 0.36 | - | 26 | 0.06 | - | - | 0.9 |
|  | All | Mean | 2.6 | 3.8 | 2.1 | 0.81 | <0.1 | 3260 | 1.6 | <2 | <0.03 | 3.2 |
|  |  | SD | 5.5 | 4.6 | 4.0 | 0.83 | - | 5400 | 1.8 | - | - | 2.1 |
| **S** | **A** | Mean | 4.1 | 27.6 | 2.92 | 15.3 | 1.32 | <200 | 9.86 | 22.7 | 0.637 | 33.8 |
|  |  | SD | 0.3 | 0.8 | 0.08 | 1.2 | 0.12 | - | 0.43 | 1.1 | 0.019 | 0.5 |
|  | **B** | Mean | <1 | 1.16 | 0.36 | <0.5 | <0.1 | 744 | 0.49 | <2 | <0.03 | 7.3 |
|  |  | SD | - | 0.31 | 0.01 | - | - | 16 | 0.09 | - | - | 0.3 |
|  | **C** | Mean | 3.1 | 9.6 | 1.71 | 10.2 | 0.69 | <200 | 3.44 | 15.5 | 0.181 | 18.2 |
|  |  | SD | 0.1 | 1.4 | 0.10 | 0.01 | 0.09 | - | 0.35 | 0.6 | 0.025 | 2.2 |
|  | **D** | Mean | <1 | 3.44 | 1.51 | 2.02 | 0.30 | 2240 | 1.47 | 5.8 | <0.03 | 16.7 |
|  |  | SD | - | 0.01 | 0.26 | 0.49 | 0.03 | 300 | 0.15 | 2.5 | - | 2.0 |
|  | **E** | Mean | 3.8 | 8.78 | 3.77 | 4.44 | 0.30 | <200 | 2.80 | <2 | 0.255 | 22.1 |
|  |  | SD | 0.4 | 0.20 | 0.12 | 1.50 | 0.03 | - | 0.09 | - | 0.005 | 0.1 |
|  | **F** | Mean | <1 | 0.40 | <0.2 | <0.5 | <0.1 | <200 | <0.2 | <2 | <0.03 | 7.8 |
|  |  | SD | - | 0.21 | - | - | - | - | - | - | - | 0.2 |
|  | **G** | Mean | 1.7 | 12.0 | 2.59 | 7.06 | 0.80 | <200 | 4.19 | 11.6 | 0.175 | 17.3 |
|  |  | SD | 0.7 | 2.6 | 0.55 | 2.32 | 0.31 | - | 0.96 | 3.7 | 0.047 | 6.9 |
|  | **H** | Mean | <1 | 3.76 | 0.50 | 2.80 | 0.28 | <200 | 1.41 | 3.5 | <0.03 | 7.7 |
|  |  | SD | - | 0.26 | 0.06 | 0.69 | 0.08 | - | 0.20 | 3.5 | - | 2.2 |
|  | All | Mean | 1.9 | 8.3 | 1.7 | 5.3 | 0.47 | 448 | 3.0 | 7.9 | 0.16 | 16.4 |
|  |  | SD | 1.5 | 8.6 | 1.3 | 5.2 | 0.43 | 736 | 3.0 | 7.9 | 0.21 | 8.9 |

^a^ LOD, limit of determination.

| **P_A** | **P_B** |
| --- | --- |
| **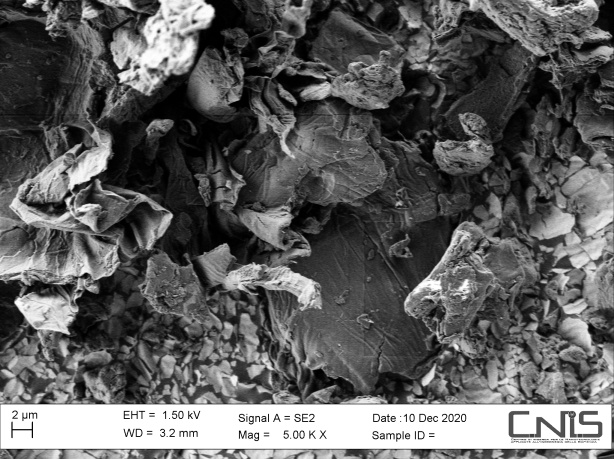** | **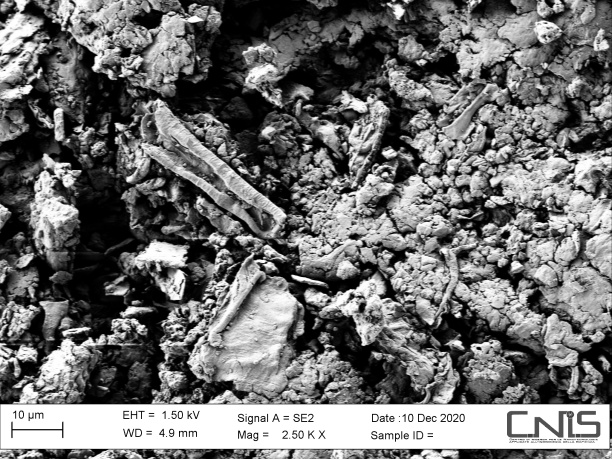** |
| **P_C** | **P_D** |
| **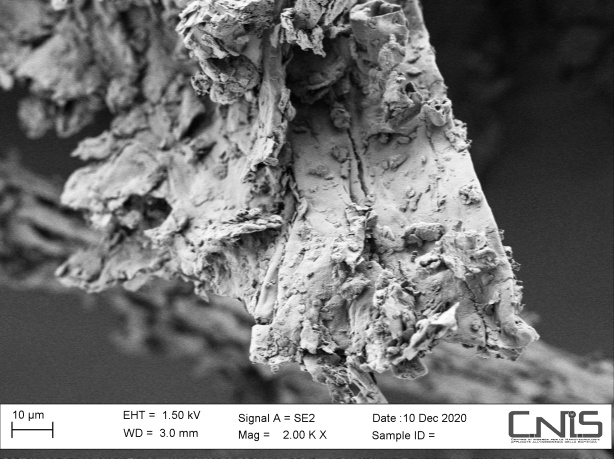** | **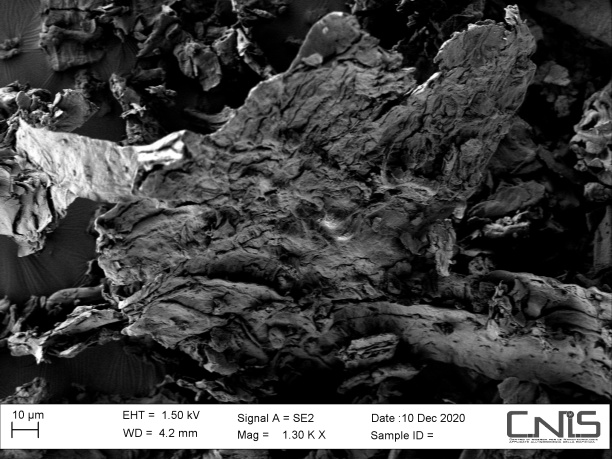** |
| **P_E** | **P_F** |
| **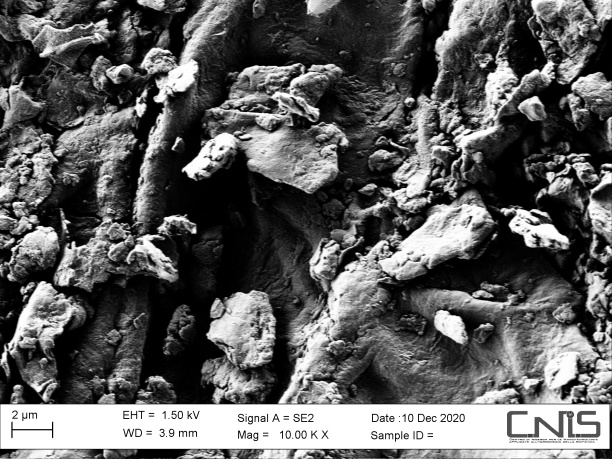** | **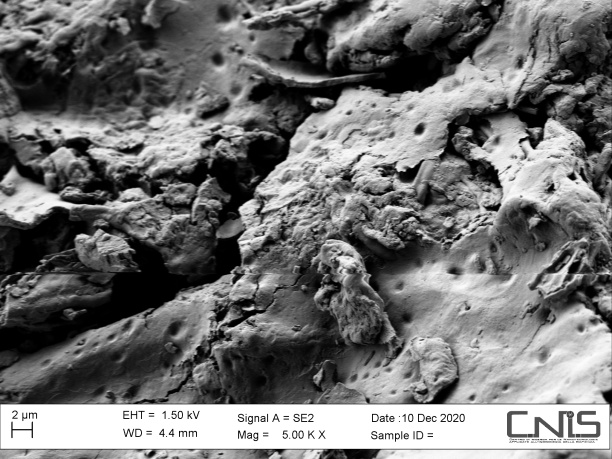** |
| **P_G** | **P_H** |
| **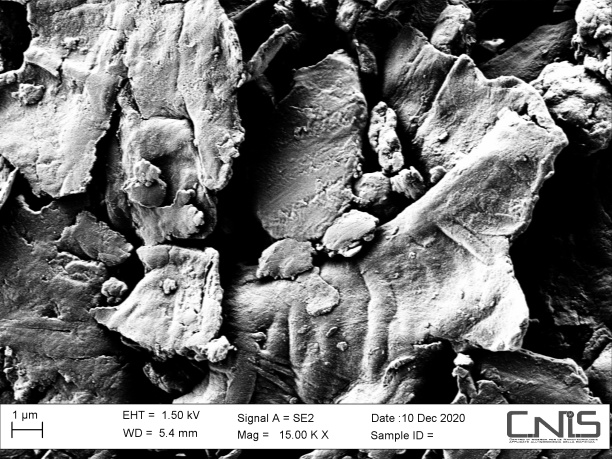** | **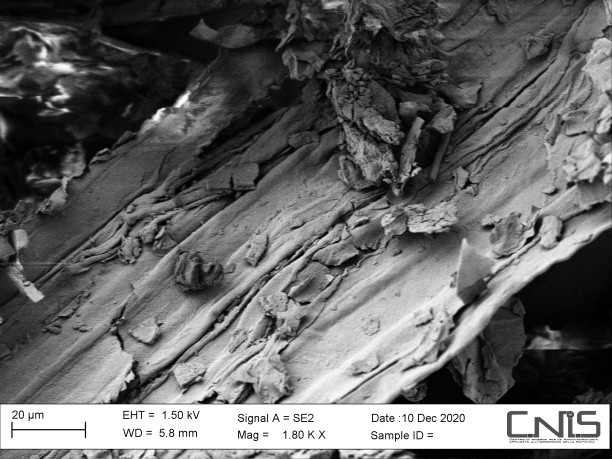** |

**Fig. S1.** Micrographs (from 1 to 20 µm) of peat (P) surface from each site (A, B, C, D, E, F, G, H) obtained by HRFESEM.

| **S_A** | **S_B** |
| --- | --- |
| **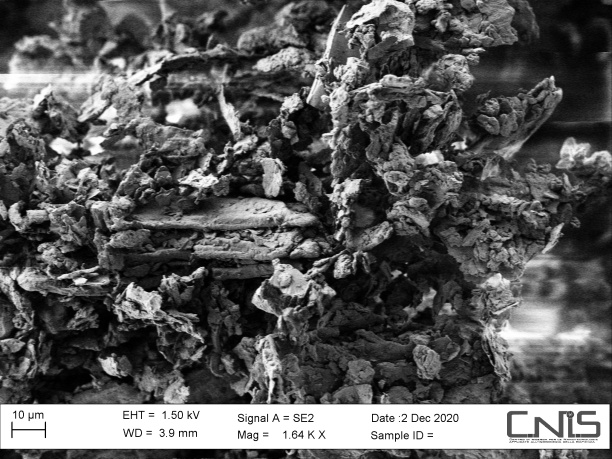** | **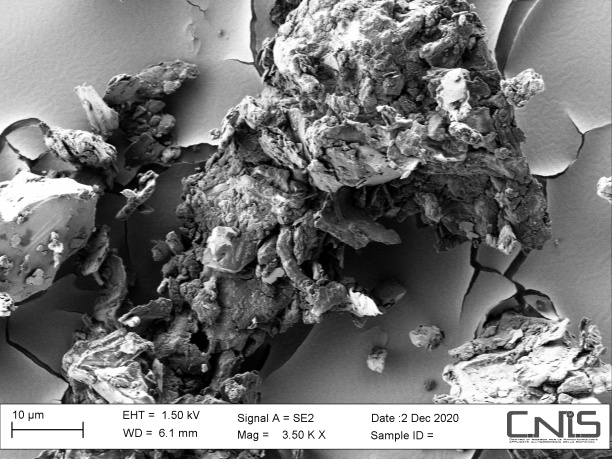** |
| **S_C** | **S_D** |
| **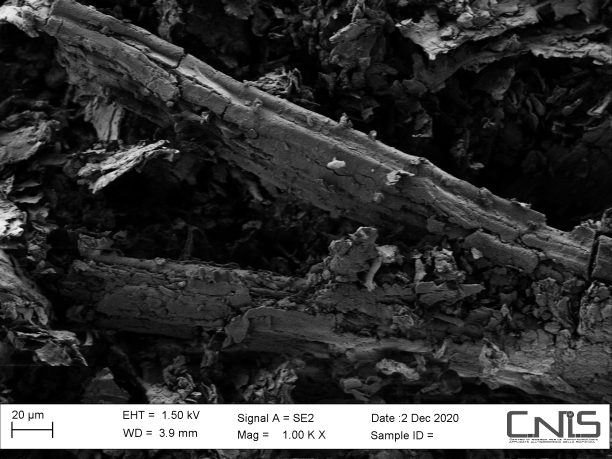** | **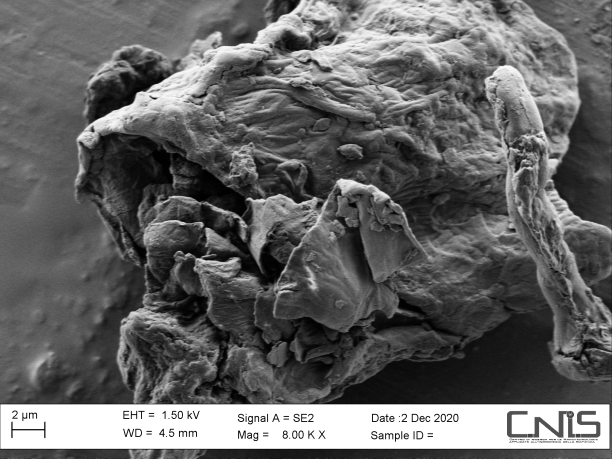** |
| **S_E** | **S_F** |
| **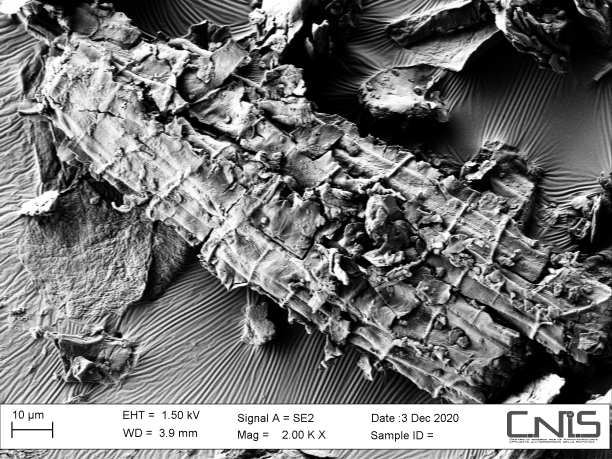** | **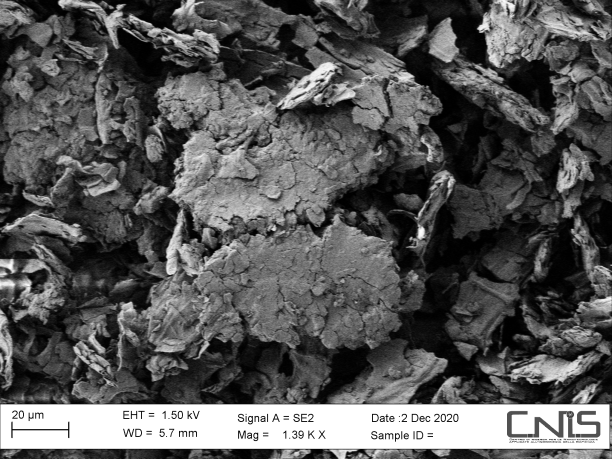** |
| **S_G** | **S_H** |
| **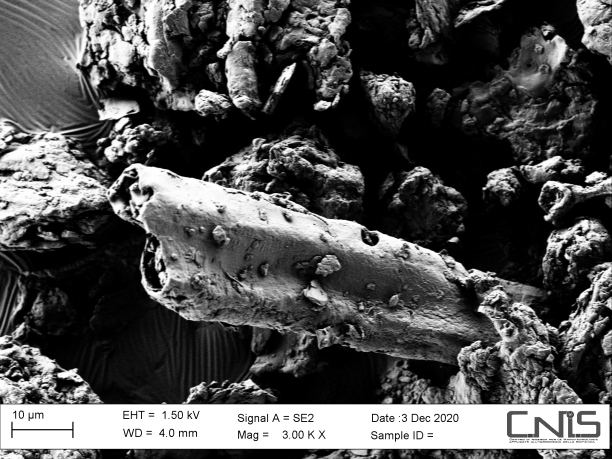** | **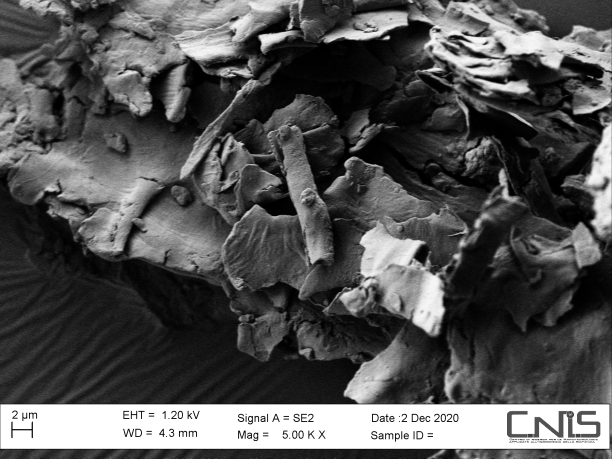** |

**Fig. S2.** Micrographs (from 2 to 20 µm) of *Sphagnum* moss (S) surface from each site (A, B, C, D, E, F, G, H) obtained by HRFESEM.

|   A |
| --- |
|   B |

**Fig. S3.** Two typical spectra of peat samples obtained by XEDS microanalysis.

**Table S3.** Elemental percentage contents (wt.%) in peat samples (range, minimum - maximum) by XEDS microanalysis (n=16).

| **Element** | **Minimum** | **Error (1σ)** | **Maximum** | **Error (1σ)** |
| --- | --- | --- | --- | --- |
| Al | 0.04 | 0.03 | 0.35 | 0.06 |
| Ca | 0.03 | 0.03 | 16.9 | 0.5 |
| Cl | 0.01 | 0.03 | 0.27 | 0.04 |
| Cr | - | - | 12.0 | 0.3 |
| Cu | - | - | 2.80 | 0.13 |
| Mg | 0.03 | 0.03 | 0.14 | 0.04 |
| Na | 0.01 | 0.03 | 0.24 | 0.05 |
| O | 25.8 | 3.7 | 45.6 | 5.3 |
| P | 0.01 | 0.03 | 0.06 | 0.03 |
| S | 0.12 | 0.03 | 0.99 | 0.08 |
| Si | 0.08 | 0.03 | 1.33 | 0.11 |

|   A |
| --- |
|   B |

**Fig. S4.** Two typical spectra of *Sphagnum* moss sample obtained by XEDS microanalysis.

**Table S4.** Elemental percentage contents in *Sphagnum* moss samples (range, minimum - maximum) by XEDS microanalysis (n=16).

| **Element** | **Minimum** | **Error (1σ)** | **Maximum** | **Error (1σ)** |
| --- | --- | --- | --- | --- |
| Al | 0.01 | 0.03 | 2.31 | 0.07 |
| Ca | 0.09 | 0.03 | 2.03 | 0.07 |
| Cl | 0.02 | 0.03 | 0.23 | 0.04 |
| Fe | 0.01 | 0.03 | 3.52 | 0.07 |
| K | 0.09 | 0.03 | 1.36 | 0.07 |
| Mg | 0.08 | 0.03 | 0.78 | 0.08 |
| N | 0.41 | 0.13 | 3.85 | 0.71 |
| Na | 0.03 | 0.03 | 0.48 | 0.05 |
| O | 29.7 | 3.7 | 44.5 | 5.3 |
| P | 0.02 | 0.03 | 0.35 | 0.04 |
| S | 0.01 | 0.03 | 0.41 | 0.04 |
| Si | 0.13 | 0.04 | 7.44 | 0.13 |

| 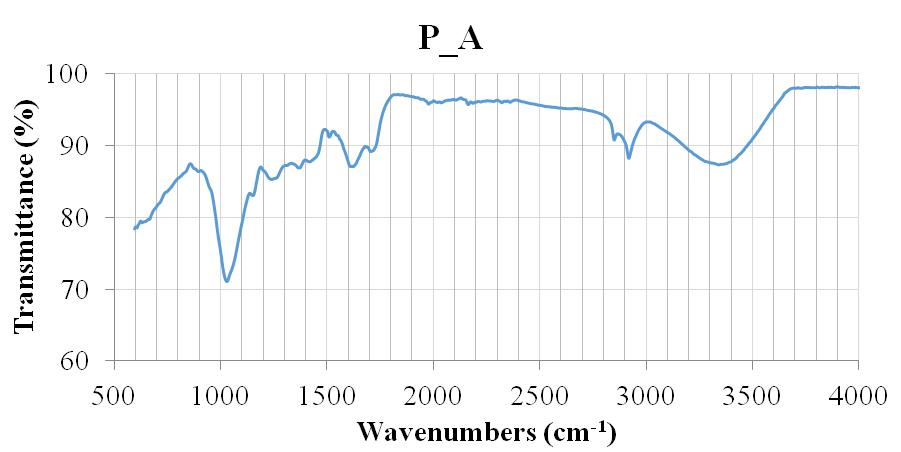 | 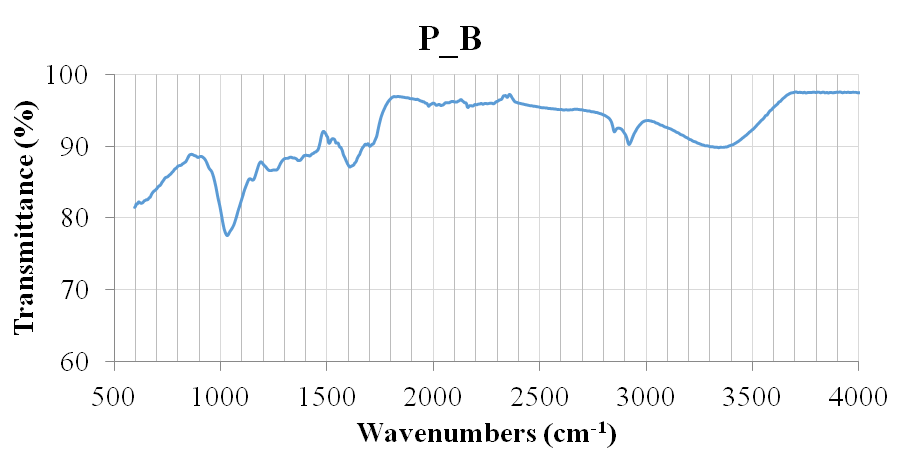 |
| --- | --- |
| 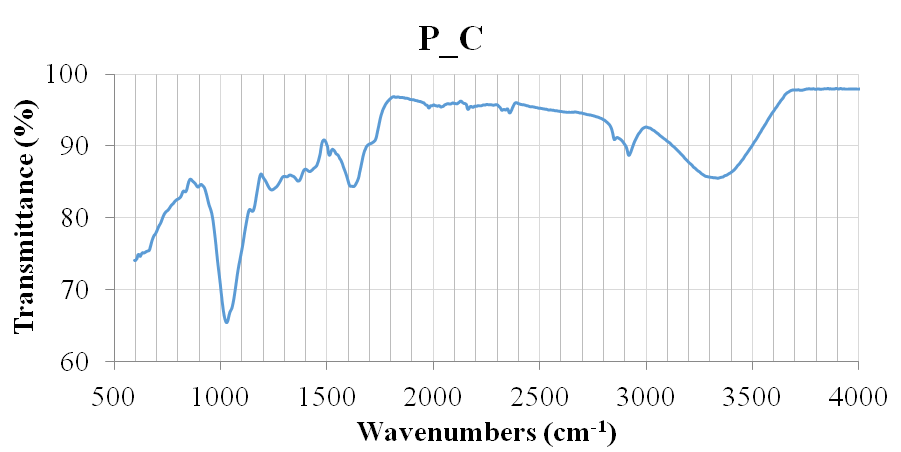 | 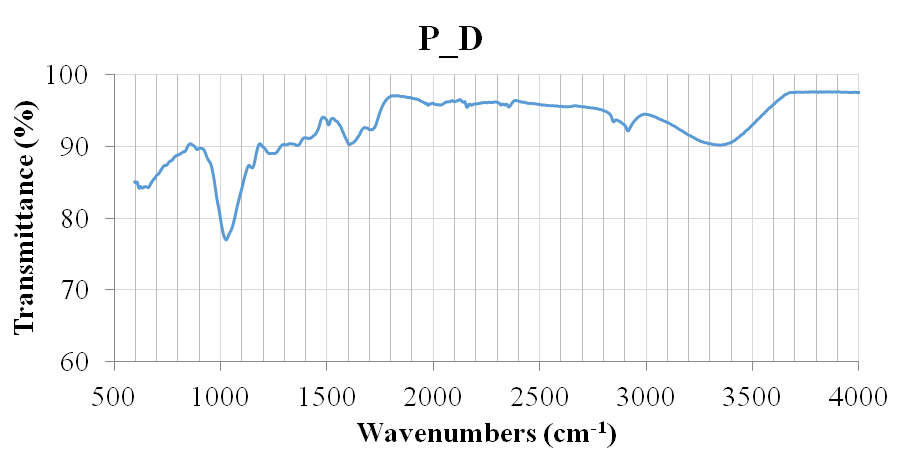 |
| 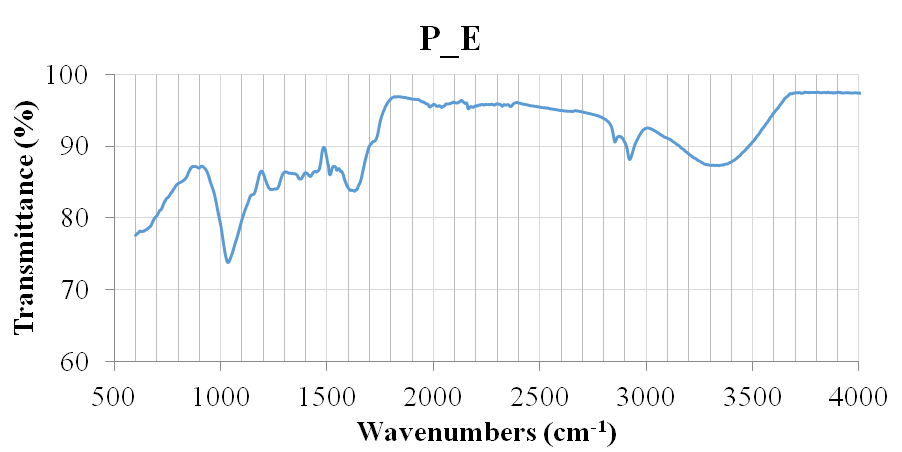 | 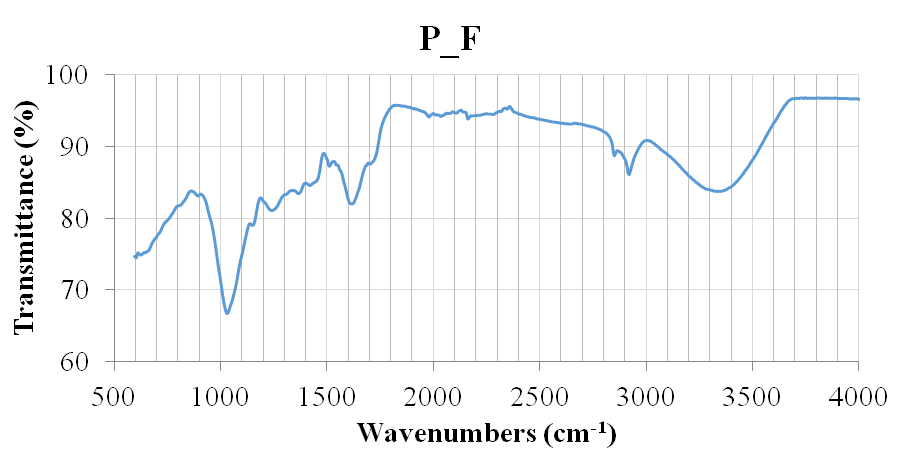 |
| 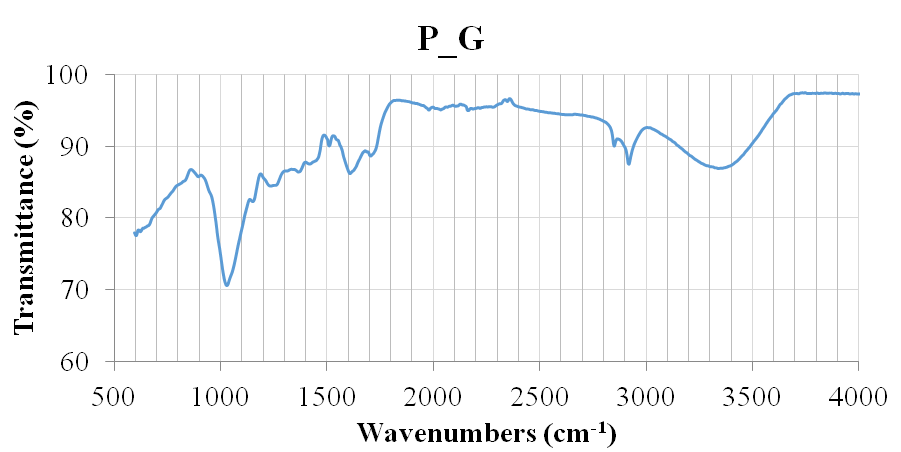 | 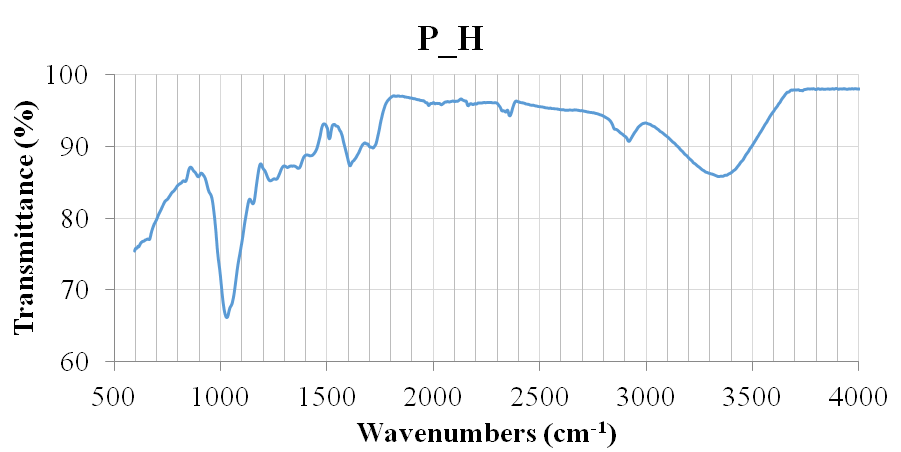 |

**Fig. S5.** FTIR spectra of peat (P) from all sites (A, B, C, D, E, F, G, and H).

| 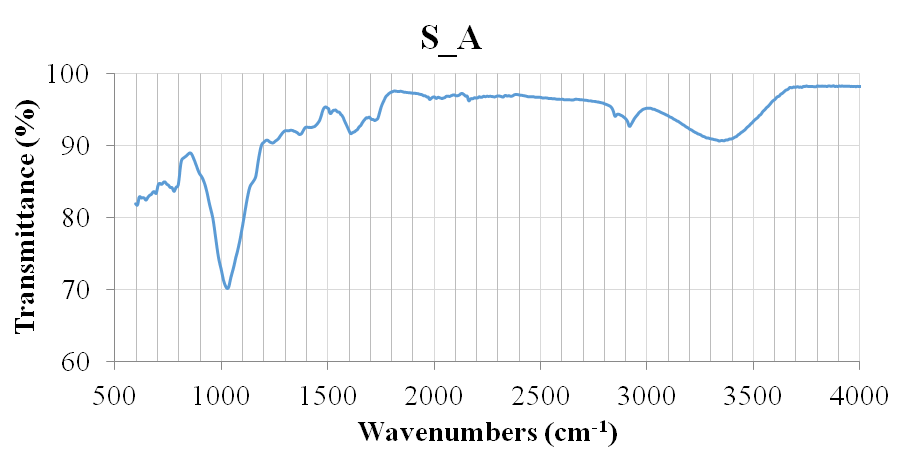 | 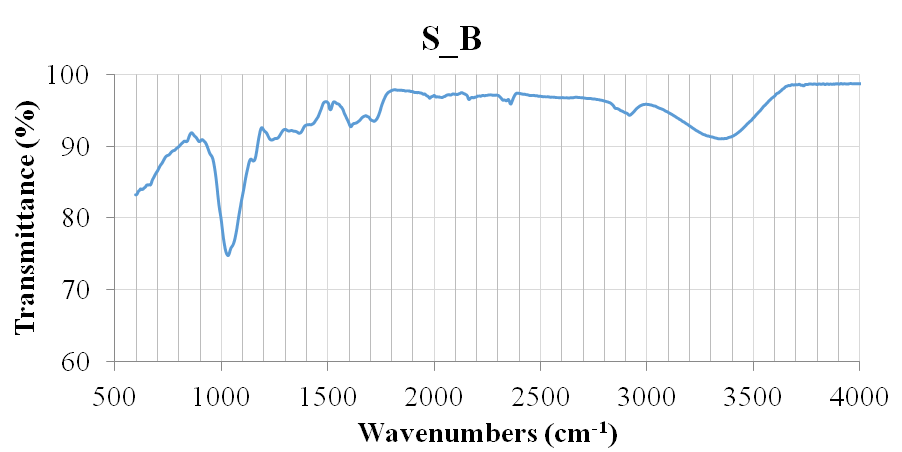 |
| --- | --- |
| 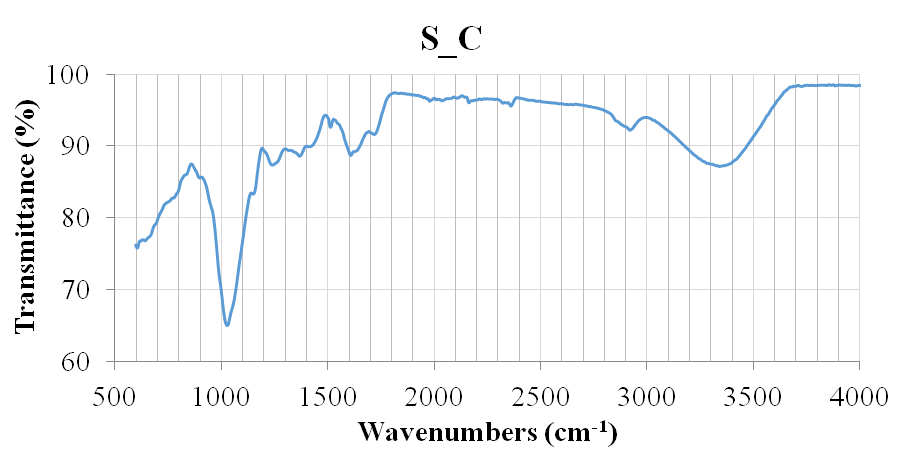 | 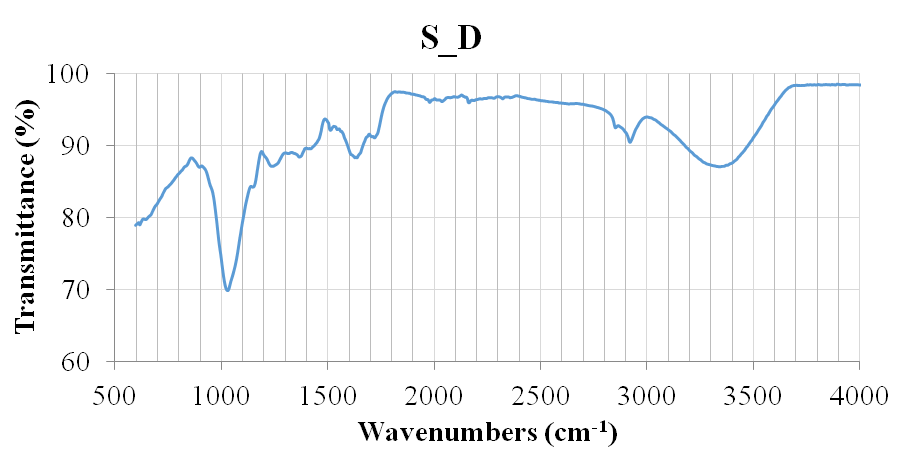 |
| 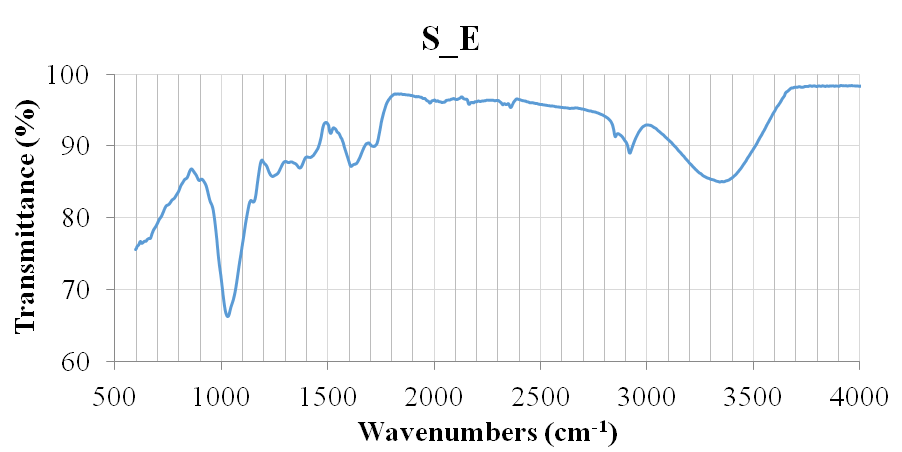 | 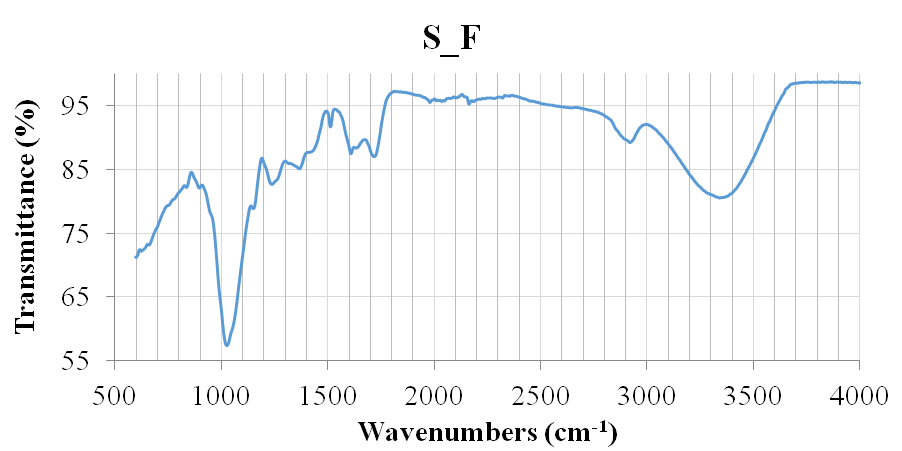 |
| 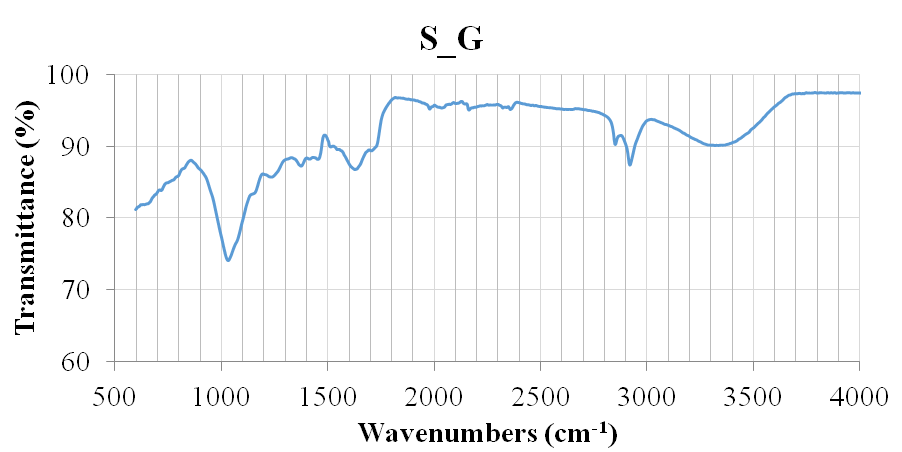 | 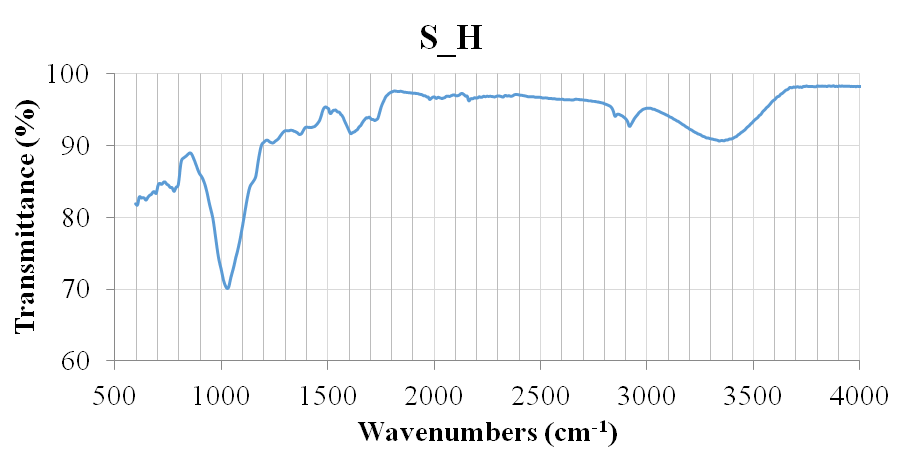 |

**Fig. S6.** FTIR spectra of *Sphagnum* moss (S) from all sites (A, B, C, D, E, F, G, and H).

**Table S5.** Main IR absorption bands in peat samples with associated functional groups.

| **Wavenumber (cm^-1^)** | **Functional groups; characterization** |
| --- | --- |
| 3,440-3,320 | ˗O-H stretching; cellulose^1,2^ |
| 2,920 | Antisymmetric CH_2_; fats, wax, lipids^1,3,4^ |
| 2,850 | Symmetric CH_2_; fats, wax, lipids^1,3,4^ |
| 1,720 | C=O stretch of COOH or COOR; carboxylic acids, aromatic esters^1,3,5,6^ |
| 1,710-1,707 | C=O stretch of COOH; free organic acids^6^ |
| 1,653 | Amide I (C=O); proteinaceous origin^7,8^ |
| 1,650-1,600 | Aromatic C=C stretching and/or asymmetric C-O stretch in COO-, NH bend; lignin and other aromatics, or aromatic or aliphatic carboxylates^1,3,9^ |
| 1,626 | Aromatic C=C stretching^4^ |
| 1,550 | Amide II (N-H in plane); proteinaceous origin^7,8^ |
| 1,515-1,513 | Aromatic C=C stretching; lignin/phenolic backbone^1^ |
| 1,426 | Symmetric C-O stretch from COO- and OH deformation (COOH); carboxylate, carboxylic structures (humic acids)^10^ |
| 1,450, 1,370 | C-H deformations; phenolic (lignin) and aliphatic structures^10^ |
| 1,265 | Aromatic CO- and phenolic -OH stretching; lignin^11,12^ |
| 1,237 | C-N; secondary amides^13^ |
| 1,160 | Polysaccharides, alcoholic groups^14,15^ |
| 1,100 | Amide III, carbohydrates, aromatic ethers, Si-O-C groups^16^ |
| ~1,080 | Si-O stretching^4,17^ |
| 1,080-1,030 | combination of C-O stretching and O-H deformation; polysaccharides^12,18,19,20,21^ |
| 1,000 | Antisymmetric stretching vibration of Si-O^22,23^ |
| 916 | Clay minerals; kaolinite doublet and smectite^15^ |
| 835 | Aromatic CH out of plane; lignin^8^ |
| 698, 770-790 | Si-O-H vibrations; clays, quartz^24,25^ |

**Table S6.** Results of the total elemental content [mean and standard deviation (SD); mg kg^-1^ d.w.] in peat for each site by AMA, ICP-MS, ICP-OES, and INAA (n=16).

|  | **Site** | | | | | | | | | | | | | | | |  |
| --- | --- | --- | --- | --- | --- | --- | --- | --- | --- | --- | --- | --- | --- | --- | --- | --- | --- |
|  | **A** | | **B** | | **C** | | **D** | | **E** | | **F** | | **G** | | **H** | |  |
| **Element** | **Mean** | **SD** | **Mean** | **SD** | **Mean** | **SD** | **Mean** | **SD** | **Mean** | **SD** | **Mean** | **SD** | **Mean** | **SD** | **Mean** | **SD** | **p-value** |
| **Al** | 1,170 | 320 | 2,120 | 93 | 1,700 | 160 | 752 | 170 | 1,450 | 400 | 438 | 41 | 594 | 45 | 536 | 45 | ns |
| **As** | 0.37 | 0.23 | 1.1 | 0.2 | 0.42 | 0.13 | 0.46 | 0.05 | 13.0 | 1.0 | 0.37 | 0.17 | <0.2 | 0.11 | <0.2 | 0.14 | ns |
| **B** | <5 | - | <5 | - | <5 | - | <5 | - | <5 | - | 7.3 | 2.7 | <5 | - | <5 | - | - |
| **Ba** | 25.9 | 6.5 | 57.1 | 7.2 | 27.5 | 0.6 | 15.2 | 1.6 | 85.2 | 14 | 11.8 | 1.7 | 20.7 | 4.6 | 9.2 | 0.2 | ns |
| **Be** | 0.027 | 0.008 | 0.100 | 0.006 | 0.060 | 0.003 | 0.030 | 0.002 | 0.166 | 0.019 | <0.02 | - | 0.030 | 0.003 | <0.02 | - | ns |
| **Bi** | <0.01 | - | <0.01 | - | 0.011 | 0.002 | <0.01 | - | 0.015 | 0.002 | <0.01 | - | <0.01 | - | <0.01 | - | - |
| **Ca** | 1,120 | 200 | 3,770 | 240 | 3,300 | 20 | 760 | 80 | 2,530 | 410 | 3,180 | 180 | 1,190 | 46 | 796 | 32 | ns |
| **Cd** | 0.072 | 0.002 | 0.133 | 0.031 | 0.064 | 0.003 | 0.042 | 0.001 | 0.149 | 0.026 | 0.045 | 0.002 | 0.036 | 0.002 | 0.050 | 0.002 | ns |
| **Ce** | 1.58 | 0.30 | 7.93 | 0.98 | 2.07 | 0.08 | 1.25 | 0.14 | 10.5 | 0.6 | 0.47 | 0.03 | 1.12 | 0.03 | 0.46 | 0.12 | ns |
| **Co** | 0.45 | 0.02 | 1.18 | 0.03 | 0.58 | 0.06 | 0.57 | 0.10 | 9.64 | 0.38 | <0.4 | - | 0.45 | 0.01 | <0.4 | - | ns |
| **Cr** | 2.4 | 1.1 | 1.5 | 1.5 | 2.28 | 0.25 | 1.96 | 0.50 | 2.76 | 0.69 | 6.4 | 4.1 | 3.56 | 0.65 | 4.88 | 0.16 | ns |
| **Cs** | 0.044 | 0.011 | 0.029 | 0.006 | 0.091 | 0.011 | 0.064 | 0.002 | 0.066 | 0.004 | 0.016 | 0.004 | 0.044 | 0.004 | 0.021 | 0.004 | ns |
| **Cu** | 1.86 | 0.24 | 6.03 | 0.24 | 2.37 | 0.09 | 1.38 | 0.19 | 4.33 | 0.75 | 2.18 | 0.50 | 1.69 | 0.06 | 0.89 | 0.11 | ns |
| **Eu** | <0.06 | - | 0.19 | 0.01 | <0.06 | - | <0.06 | - | 0.36 | 0.03 | <0.06 | - | <0.06 | - | <0.06 | - | - |
| **Fe** | 731 | 36 | 3,360 | 190 | 1,150 | 31 | 1,230 | 160 | 13,000 | 240 | 603 | 18 | 870 | 1 | 406 | 39 | ns |
| **Ga** | 0.375 | 0.094 | 0.458 | 0.002 | 0.478 | 0.055 | 0.252 | 0.005 | 0.246 | 0.026 | 0.130 | 0.025 | 0.303 | 0.002 | 0.147 | 0.021 | ns |
| **Hf** | 4.8 | 3.8 | 3.8 | 2.6 | 2.5 | 1.9 | 2.4 | 0.1 | 9.4 | 9.6 | 1.0 | 0.7 | 2.2 | 0.1 | 5.4 | 1.7 | ns |
| **Hg** | 0.0744 | 0.0026 | 0.0274 | 0.0034 | 0.0719 | 0.0045 | 0.0299 | 0.0026 | 0.0382 | 0.0040 | 0.0601 | 0.0031 | 0.0284 | 0.0009 | 0.0198 | 0.0009 | ns |
| **K** | 324 | 120 | 207 | 31 | 397 | 28 | 243 | 46 | 177 | 50 | 155 | 29 | 159 | 7 | 101 | 9 | ns |
| **La** | 0.791 | 0.147 | 3.84 | 0.42 | 0.923 | 0.033 | 0.577 | 0.046 | 3.67 | 0.35 | 0.194 | 0.022 | 0.501 | 0.003 | 0.226 | 0.052 | ns |
| **Li** | 0.148 | 0.017 | 0.165 | 0.016 | 0.185 | 0.025 | 0.048 | 0.003 | 0.122 | 0.019 | 0.058 | 0.025 | 0.254 | 0.001 | 0.027 | 0.009 | ns |
| **Lu** | 0.024 | 0.012 | 0.055 | 0.006 | 0.016 | 0.016 | <0.01 | - | 0.115 | 0.001 | <0.01 | - | <0.01 | - | <0.01 | - | ns |
| **Mg** | 984 | 7 | 526 | 40 | 689 | 18 | 1,240 | 21 | 565 | 25 | 1,400 | 10 | 130 | 55 | 916 | 14 | ns |
| **Mn** | 25.3 | 5.2 | 33.4 | 0.8 | 15.2 | 1.5 | 6.6 | 3.7 | 733 | 8 | 33.6 | 3.5 | 23.1 | 1.0 | 5.5 | 0.5 | ns |
| **Mo** | 0.18 | 0.03 | 0.44 | 0.02 | <0.1 | - | <0.1 | - | 0.17 | 0.03 | 0.64 | 0.01 | <0.1 | - | <0.1 | - | ns |
| **Na** | 665 | 44 | 399 | 44 | 564 | 49 | 306 | 40 | 255 | 66 | 542 | 24 | 401 | 14 | 176 | 13 | ns |
| **Nb** | 0.158 | 0.058 | 0.259 | 0.001 | 0.291 | 0.028 | 0.137 | 0.010 | 0.171 | 0.018 | 0.055 | 0.015 | 0.137 | 0.003 | 0.057 | 0.007 | ns |
| **Ni** | 0.70 | 0.11 | 1.87 | 0.10 | 1.22 | 0.13 | 0.68 | 0.11 | 7.44 | 0.48 | 0.42 | 0.11 | 0.73 | 0.04 | 0.55 | 0.07 | ns |
| **P** | 149 | 16 | 370 | 74 | 299 | 12 | 113 | 23 | 573 | 65 | 304 | 24 | 142 | 4 | 62 | 2 | ns |
| **Pb** | 0.96 | 0.11 | 0.81 | 0.12 | 1.19 | 0.12 | 0.63 | 0.03 | 0.71 | 0.21 | 0.26 | 0.05 | 0.61 | 0.02 | 0.52 | 0.04 | ns |
| **Rb** | 0.87 | 0.24 | 0.54 | 0.12 | 1.00 | 0.22 | 0.59 | 0.02 | 1.72 | 0.04 | 0.36 | 0.06 | 0.57 | 0.08 | 0.28 | 0.04 | ns |
| **S** | 1,720 | 280 | 2,870 | 190 | 3,260 | 91 | 1,450 | 160 | 2,320 | 300 | 2,190 | 5 | 2,450 | 140 | 1,290 | 97 | ns |
| **Sb** | <0.2 | - | <0.2 | - | <0.2 | - | <0.2 | - | <0.2 | - | <0.2 | - | <0.2 | - | <0.2 | - | - |
| **Sc** | 0.79 | 0.16 | 1.13 | 0.09 | 0.94 | 0.13 | 0.390 | 0.044 | 1.34 | 0.23 | 0.162 | 0.024 | 0.453 | 0.005 | 0.157 | 0.009 | ns |
| **Se** | <0.5 | - | 0.77 | 0.09 | 0.48 | 0.14 | 0.67 | 0.49 | <0.5 | - | <0.5 | - | 0.94 | 0.04 | <0.5 | - | - |
| **Si** | 6,860 | 2,400 | 3,680 | 1,140 | 7,440 | 560 | 3,340 | 860 | 2,700 | 840 | 2,230 | 100 | 3,200 | 140 | 1,200 | 160 | ns |
| **Sm** | 0.200 | 0.036 | 0.87 | 0.12 | 0.275 | 0.040 | 0.129 | 0.004 | 1.66 | 0.13 | 0.066 | 0.013 | 0.106 | 0.011 | 0.059 | 0.006 | ns |
| **Sn** | 0.103 | 0.015 | 0.041 | 0.014 | 0.075 | 0.005 | 0.061 | 0.009 | 0.032 | 0.005 | 0.034 | 0.008 | 0.051 | 0.025 | <0.02 | - | ns |
| **Sr** | 21.2 | 1.9 | 52.5 | 0.2 | 15.8 | 0.8 | 23.7 | 1.5 | 52.9 | 1.9 | 36.2 | 1.2 | 21.1 | 0.5 | 14.3 | 0.9 | ns |
| **Te** | <0.02 | - | <0.02 | - | <0.02 | - | <0.02 | - | <0.02 | - | <0.02 | - | <0.02 | - | <0.02 | - | - |
| **Th** | 0.281 | 0.052 | 0.755 | 0.078 | 0.521 | 0.058 | 0.269 | 0.014 | 1.68 | 0.07 | 0.12 | 0.14 | 0.256 | 0.007 | 0.075 | 0.070 | ns |
| **Ti** | 125 | 45 | 98.8 | 7.5 | 158 | 15 | 65 | 12 | 45 | 14 | 25.2 | 4.0 | 74.8 | 0.2 | 28.3 | 5.0 | ns |
| **Tl** | 0.008 | 0.001 | 0.007 | 0.000 | 0.031 | 0.001 | 0.009 | 0.002 | 0.031 | 0.002 | 0.007 | 0.001 | 0.009 | 0.000 | 0.005 | 0.002 | ns |
| **U** | 0.093 | 0.027 | 0.163 | 0.004 | 0.083 | 0.003 | 0.039 | 0.004 | 0.121 | 0.020 | 0.025 | 0.002 | 0.046 | 0.003 | 0.017 | 0.002 | ns |
| **V** | 2.62 | 0.80 | 4.21 | 0.47 | 3.60 | 0.01 | 1.50 | 0.20 | 3.00 | 0.11 | 0.74 | 0.16 | 2.22 | 0.22 | 0.75 | 0.07 | ns |
| **W** | <0.1 | - | <0.1 | - | <0.1 | - | <0.1 | - | <0.1 | - | <0.1 | - | <0.1 | - | <0.1 | - | - |
| **Yb** | 0.14 | 0.04 | 0.34 | 0.05 | <0.1 | - | <0.1 | - | 0.86 | 0.08 | <0.1 | - | <0.1 | - | <0.1 | - | - |
| **Zn** | <1 | - | 2.4 | 5.3 | <1 | - | <1 | - | 6.2 | 0.7 | <1 | - | 2.1 | 0.4 | <1 | - | ns |
| **Zr** | 8,950 | 7,800 | 4,500 | 2,900 | 7,290 | 5,200 | 4,480 | 250 | 17,300 | 18,000 | 1,740 | 1,300 | 3,790 | 1,200 | 10,900 | 3,300 | ns |

^a^ Non-parametric Kruskal-Wallis test was applied: “-“ = not determined; “ns” = not significant at p >0.05.

**Table S7.** Results of the total elemental content [mean and standard deviation (SD); mg kg^-1^ d.w.] in *Sphagnum* moss for each site by AMA, ICP-MS, ICP-OES, and INAA (n=16).

|  | **Site** | | | | | | | | | | | | | | | |  |
| --- | --- | --- | --- | --- | --- | --- | --- | --- | --- | --- | --- | --- | --- | --- | --- | --- | --- |
|  | **A** | | **B** | | **C** | | **D** | | **E** | | **F** | | **G** | | **H** | |  |
| **Element** | **Mean** | **SD** | **Mean** | **SD** | **Mean** | **SD** | **Mean** | **SD** | **Mean** | **SD** | **Mean** | **SD** | **Mean** | **SD** | **Mean** | **SD** | **p-value** |
| **Al** | 7,780 | 12 | 633 | 10 | 1,590 | 30 | 1,180 | 440 | 1,390 | 290 | 296 | 45 | 5,100 | 600 | 1,170 | 2 | ns |
| **As** | 2.67 | 0.27 | 0.32 | 0.03 | 2.03 | 0.42 | 0.46 | 0.10 | 2.56 | 0.20 | <0.2 | 0.11 | 1.25 | 0.01 | 0.47 | 0.07 | ns |
| **B** | <5 | - | <5 | - | <5 | - | <5 | - | <5 | - | <5 | - | <5 | - | <5 | - | - |
| **Ba** | 80.7 | 2.3 | 22.2 | 0.7 | 61.5 | 6.2 | 24.9 | 1.6 | 32.1 | 6.0 | 3.3 | 0.8 | 67 | 15 | 27.0 | 0.1 | ns |
| **Be** | 0.160 | 0.030 | <0.02 | - | 0.086 | 0.005 | 0.026 | 0.001 | 0.060 | 0.002 | <0.02 | - | 0.154 | 0.025 | 0.020 | 0.002 | ns |
| **Bi** | 0.062 | 0.007 | <0.01 | - | 0.022 | 0.001 | 0.006 | 0.001 | 0.031 | 0.004 | <0.01 | - | 0.037 | 0.009 | <0.01 | - | - |
| **Ca** | 1,940 | 48 | 2,800 | 28 | 3,190 | 240 | 1,040 | 29 | 1,920 | 290 | 1,260 | 180 | 5,960 | 340 | 2,680 | 79 | ns |
| **Cd** | 0.259 | 0.037 | 0.033 | 0.010 | 0.140 | 0.017 | 0.206 | 0.017 | 0.165 | 0.051 | 0.032 | 0.003 | 0.144 | 0.017 | 0.058 | 0.005 | ns |
| **Ce** | 18.4 | 2.8 | 1.15 | 0.24 | 6.87 | 0.27 | 3.16 | 0.20 | 6.42 | 0.21 | 0.20 | 0.02 | 9.14 | 2.28 | 1.62 | 0.07 | ns |
| **Co** | 5.24 | 0.10 | 0.53 | 0.01 | 2.64 | 0.29 | 1.74 | 0.22 | 3.41 | 0.65 | <0.4 | - | 3.26 | 0.70 | 0.50 | 0.07 | ns |
| **Cr** | 11.7 | 0.53 | 0.47 | 0.07 | 7.7 | 1.3 | 1.41 | 0.04 | 2.08 | 0.58 | 0.11 | 0.19 | 4.7 | 1.2 | 1.18 | 0.14 | ns |
| **Cs** | 0.550 | 0.091 | 0.055 | 0.008 | 0.387 | 0.006 | 0.233 | 0.008 | 0.130 | 0.002 | 0.010 | 0.000 | 0.40 | 0.11 | 0.081 | 0.007 | ns |
| **Cu** | 9.25 | 0.17 | 1.15 | 0.19 | 3.24 | 0.05 | 1.82 | 0.33 | 6.01 | 0.17 | 0.59 | 0.03 | 5.5 | 1.2 | 1.24 | 0.03 | ns |
| **Eu** | 0.47 | 0.06 | <0.06 | - | 0.15 | 0.01 | <0.06 | - | 0.16 | 0.01 | <0.06 | - | 0.21 | 0.04 | 0.07 | 0.01 | - |
| **Fe** | 7,680 | 470 | 734 | 140 | 4,630 | 5 | 1,920 | 104 | 5,840 | 180 | 125 | 2 | 4,840 | 1,400 | 781 | 31 | ns |
| **Ga** | 2.57 | 0.30 | 0.207 | 0.033 | 1.34 | 0.06 | 0.339 | 0.030 | 0.735 | 0.002 | 0.048 | 0.003 | 1.36 | 0.32 | 0.316 | 0.023 | ns |
| **Hf** | 58.7 | 29.8 | 2.8 | 0.3 | 13.7 | 7.7 | 6.0 | 0.5 | 22.8 | 15.2 | 11.6 | 4.0 | 7.4 | 5.2 | 16.7 | 4.4 | ns |
| **Hg** | 0.0720 | 0.0057 | 0.0417 | 0.0001 | 0.0417 | 0.0001 | 0.101 | 0.009 | 0.0706 | 0.0015 | 0.0239 | 0.0020 | 0.115 | 0.001 | 0.0380 | 0.0038 | ns |
| **K** | 3,560 | 16 | 690 | 4 | 2,640 | 230 | 1,300 | 96 | 944 | 200 | 1,730 | 270 | 1,800 | 600 | 1,200 | 100 | ns |
| **La** | 7.62 | 1.19 | 0.424 | 0.095 | 2.61 | 0.10 | 1.08 | 0.01 | 2.19 | 0.07 | 0.076 | 0.007 | 3.40 | 0.78 | 0.616 | 0.031 | ns |
| **Li** | 3.71 | 0.26 | 0.487 | 0.046 | 1.64 | 0.07 | 0.189 | 0.066 | 0.703 | 0.054 | 0.034 | 0.007 | 2.2 | 1.2 | 0.226 | 0.014 | ns |
| **Lu** | 0.174 | 0.008 | <0.01 | - | 0.072 | 0.003 | 0.016 | 0.016 | 0.052 | 0.007 | <0.01 | - | 0.082 | 0.008 | 0.037 | 0.001 | ns |
| **Mg** | 1,920 | 29 | 1,200 | 170 | 2,400 | 98 | 1,380 | 120 | 1,340 | 41 | 446 | 19 | 1,150 | 280 | 703 | 24 | ns |
| **Mn** | 452 | 138 | 187 | 4 | 200 | 11 | 21 | 8 | 473 | 26 | 249 | 19 | 371 | 227 | 168 | 3 | ns |
| **Mo** | 0.16 | 0.01 | <0.1 | - | <0.1 | - | <0.1 | - | <0.1 | - | <0.1 | - | 0.25 | 0.05 | <0.1 | - | ns |
| **Na** | 3,630 | 62 | 726 | 15 | 1,340 | 160 | 694 | 130 | 790 | 79 | 546 | 90 | 1,350 | 380 | 713 | 8 | ns |
| **Nb** | 0.337 | 0.031 | 0.101 | 0.016 | 0.426 | 0.002 | 0.171 | 0.017 | 0.168 | 0.002 | 0.010 | 0.001 | 0.398 | 0.030 | 0.127 | 0.003 | ns |
| **Ni** | 5.27 | 0.12 | 0.53 | 0.09 | 3.29 | 0.09 | 0.92 | 0.03 | 3.39 | 0.14 | 0.09 | 0.07 | 3.86 | 0.81 | 0.66 | 0.04 | ns |
| **P** | 458 | 10 | 230 | 15 | 395 | 35 | 623 | 11 | 370 | 33 | 473 | 66 | 777 | 20 | 152 | 19 | ns |
| **Pb** | 7.48 | 0.16 | 0.732 | 0.054 | 5.47 | 0.52 | 1.56 | 0.01 | 10.2 | 0.1 | 0.140 | 0.014 | 4.3 | 1.8 | 0.873 | 0.016 | ns |
| **Rb** | 9.1 | 1.2 | 1.18 | 0.20 | 5.09 | 0.22 | 2.18 | 0.07 | 3.12 | 0.29 | 0.93 | 0.04 | 4.9 | 1.8 | 1.40 | 0.24 | ns |
| **S** | 531 | 54 | 361 | 14 | 541 | 36 | 1,190 | 36 | 537 | 66 | 276 | 53 | 2,020 | 120 | 316 | 38 | ns |
| **Sb** | <0.2 | - | <0.2 | - | <0.2 | - | <0.2 | - | <0.2 | - | <0.2 | - | <0.2 | - | <0.2 | - | - |
| **Sc** | 5.39 | 0.18 | 0.231 | 0.028 | 2.22 | 0.24 | 0.657 | 0.080 | 1.31 | 0.01 | 0.095 | 0.003 | 2.38 | 0.43 | 0.83 | 0.03 | ns |
| **Se** | <0.5 | - | <0.5 | - | <0.5 | - | <0.5 | - | <0.5 | - | <0.5 | - | <0.5 | - | <0.5 | - | - |
| **Si** | 95,800 | 6,300 | 4,340 | 290 | 29,300 | 2,900 | 7,840 | 1,500 | 15,000 | 290 | 919 | 200 | 28,800 | 10,600 | 8,770 | 680 | ns |
| **Sm** | 2.35 | 0.16 | 0.106 | 0.008 | 0.80 | 0.12 | 0.263 | 0.042 | 0.710 | 0.005 | 0.048 | 0.017 | 0.81 | 0.28 | 0.321 | 0.003 | ns |
| **Sn** | 0.407 | 0.063 | 0.065 | 0.009 | 0.217 | 0.007 | 0.071 | 0.011 | 0.197 | 0.001 | <0.02 | - | 0.189 | 0.022 | 0.050 | 0.012 | ns |
| **Sr** | 28.3 | 2.7 | 17.4 | 2.9 | 28.5 | 0.7 | 29.1 | 3.5 | 25.7 | 0.1 | 11.0 | 0.9 | 51.4 | 5.9 | 13.2 | 1.0 | ns |
| **Te** | <0.02 | - | <0.02 | - | <0.02 | - | <0.02 | - | <0.02 | - | <0.02 | - | <0.02 | - | <0.02 | - | - |
| **Th** | 2.91 | 0.13 | 0.202 | 0.001 | 1.23 | 0.19 | 0.404 | 0.071 | 1.01 | 0.04 | 0.074 | 0.069 | 1.76 | 0.28 | 0.600 | 0.014 | ns |
| **Ti** | 1,042 | 28 | 58.1 | 3.4 | 388 | 31 | 130 | 27 | 208 | 8 | 13.7 | 0.5 | 416 | 120 | 156 | 21 | ns |
| **Tl** | 0.078 | 0.014 | 0.0048 | 0.0014 | 0.0836 | 0.0017 | 0.0524 | 0.0073 | 0.0371 | 0.0007 | 0.0016 | 0.0001 | 0.056 | 0.012 | 0.0146 | 0.0010 | ns |
| **U** | 0.307 | 0.037 | 0.028 | 0.019 | 0.119 | 0.001 | 0.062 | 0.001 | 0.129 | 0.001 | 0.006 | 0.001 | 0.170 | 0.009 | 0.028 | 0.001 | ns |
| **V** | 18.4 | 3.0 | 1.18 | 0.19 | 8.21 | 0.26 | 2.12 | 0.12 | 6.08 | 0.17 | 0.38 | 0.11 | 8.26 | 1.31 | 2.07 | 0.20 | ns |
| **W** | <0.1 | - | <0.1 | - | <0.1 | - | <0.1 | - | <0.1 | - | <0.1 | - | <0.1 | - | <0.1 | - | - |
| **Yb** | 1.05 | 0.10 | <0.1 | - | 0.43 | 0.08 | <0.1 | - | 0.26 | 0.05 | <0.1 | - | 0.49 | 0.05 | 0.17 | 0.04 | - |
| **Zn** | 37.8 | 3.8 | 9.8 | 1.5 | 24.0 | 1.0 | 17.0 | 3.7 | 26.8 | 1.0 | 9.2 | 0.7 | 21.5 | 7.7 | 8.8 | 1.3 | ns |
| **Zr** | 2,200 | 1,100 | 111 | 5 | 587 | 220 | 292 | 23 | 890 | 620 | 434 | 130 | 277 | 220 | 655 | 270 | ns |

^a^ Non-parametric Kruskal-Wallis test was applied: “-“ = not determined; “ns” = not significant at p >0.05.

**Table S8.** Threshold and guideline values (mg kg^-1^) for the dangerous elements in soils.

|  | **Legislative Decree 152/2006 and** **subsequent amendments and additions** | | | **Ministry of the Environment, Finland 2007** | | |
| --- | --- | --- | --- | --- | --- | --- |
| **Elements** | **Agricultural soil contamination threshold^a^** | **Sites for public, private and residential green areas** | **Sites for commercial and industrial use** | **Threshold value** | **Lower guideline value^b^** | **Higher guideline value^b^** |
| **As** | 30 | 20 | 50 | 5 | 50 (e) | 100 (e) |
| **Be** | 7 | 2 | 10 | NG^c^ | NG^c^ | NG^c^ |
| **Cd** | 5 | 2 | 15 | 1 | 10 (e) | 20 (e) |
| **Co** | 30 | 20 | 250 | 20 | 100 (e) | 250 (e) |
| **Cr** | 150 | 150 | 800 | 100 | 200 (e) | 300 (e) |
| **Cu** | 200 | 120 | 600 | 100 | 150 (e) | 200 (e) |
| **Hg** | 1 | 1 | 5 | 0.5 | 2 (e) | 5 (e) |
| **Ni** | 120 | 120 | 500 | 50 | 100 (e) | 150 (e) |
| **Pb** | 100 | 100 | 1,000 | 60 | 200 (t) | 250 (e) |
| **Sb** | 10 | 10 | 30 | 2 | 10 (t) | 50 (e) |
| **Se** | 3 | 3 | 15 | NG^c^ | NG^c^ | NG^c^ |
| **Sn** | NG^c^ | 1 | 350 | NG^c^ | NG^c^ | NG^c^ |
| **Tl** | 1 | 1 | 10 | NG^c^ | NG^c^ | NG^c^ |
| **V** | 90 | 90 | 250 | 100 | 150 (e) | 250 (e) |
| **Zn** | 300 | 150 | 1,500 | 200 | 250 (e) | 400 (e) |

^a^ Ministerial Degree 46/2019 Annex 2, Article 3.

^b^ The guideline values have been defined on the basis of either ecological risks (e) or health risks (t).

^c^ NG stands for Not Given.

**Table S9.** Stepwise variables result for C, H, N, O and S contents.

| **Step** |  | **Variables** | **SP^a^** | **SSS^b^** | **R^2c^** | **Cp^d^** | **n^e^** | **AICc^f^** | **BIC^g^** |
| --- | --- | --- | --- | --- | --- | --- | --- | --- | --- |
| 1 |  | % C | 0.0013 | 2.145046 | 0.5363 | 36.03 | 2 | 18.9304 | 19.2481 |
| 2 |  | O/C | 0.0026 | 0.954894 | 0.7750 | 13.305 | 3 | 10.9963 | 10.4503 |
| 3 |  | H/C | 0.0705 | 0.222394 | 0.8306 | 9.5466 | 4 | 10.819 | 8.68195 |
| 4 |  | % S | 0.0824 | 0.168899 | 0.8728 | 7.1734 | 5 | 11.5657 | 6.86793 |
| 5 |  | C/N | 0.1515 | 0.098871 | 0.8975 | 6.6133 | 6 | 14.775 | 6.18313 |
| 6 |  | % H | 0.1615 | 0.084221 | 0.9186 | 6.4326 | 7 | 19.6664 | 5.27564 |
| 7 |  | % O* | 0.2977 | 0.04372 | 0.9295 | 7.3006 | 8 | 28.7885 | 5.7418 |
| 8 |  | % N | 0.6006 | 0.011609 | 0.9324 | 9 | 9 | 44.1158 | 7.84169 |
|  |  | Best | . | . | 0.9186 | 6.4326 | 7 | 19.6664 | 5.27564 |

^a^ SP, significant probability. ^b^ SSS, sequential sum of squares. ^c^ R^2^, coefficient of determination. ^e^ n, number of parameters in the model, including the intercept. ^f^ AICc, corrected akaike’s information criterion. ^g^ BIC, Bayesan information criterion.

**Table S10**. Stepwise variables result for infrared spectra.

| **Step** |  | **Variables** | **SP^a^** | **SSS^b^** | **R^2c^** | **Cp^d^** | **n^e^** | **AICc^f^** | **BIC^g^** |
| --- | --- | --- | --- | --- | --- | --- | --- | --- | --- |
| 1 |  | 1489 | 0.0343 | 1.128127 | 0.2820 | . | 2 | 25.924 | 26.2418 |
| 2 |  | 1662.6 | 0.0061 | 1.295054 | 0.6058 | . | 3 | 19.9675 | 19.4215 |
| 3 |  | 3194.1 | 0.0848 | 0.35835 | 0.6954 | . | 4 | 20.2061 | 18.0691 |
| 4 |  | 2306.9 | 0.0392 | 0.404905 | 0.7966 | . | 5 | 19.0767 | 14.3789 |
| 5 |  | 3437.2 | 0.1722 | 0.144609 | 0.8328 | . | 6 | 22.612 | 14.0201 |
| 6 |  | 1562.3 | 0.2312 | 0.103593 | 0.8587 | . | 7 | 28.4914 | 14.1007 |
| 7 |  | 3425.6 | 0.4497 | 0.041363 | 0.8690 | . | 8 | 38.7044 | 15.6577 |
| 8 |  | 1566.2 | 0.6589 | 0.01543 | 0.8729 | . | 9 | 54.2262 | 17.9521 |
| 9 |  | 1577.8 | 0.7830 | 0.006938 | 0.8746 | . | 10 | 78.0064 | 20.5049 |
| 10 |  | 1558.5 | 0.9087 | 0.001457 | 0.8750 | . | 11 | 117.96 | 23.2309 |
| 11 |  | 3190.3 | 0.9751 | 0.000138 | 0.8750 | . | 12 | 197.955 | 25.9991 |
| 12 |  | 2823.8 | 0.9889 | 3.768e-5 | 0.8750 | . | 13 | 437.954 | 28.7705 |
| 13 |  | 2962.7 | 0.9998 | 1.975e-8 | 0.8750 | . | 14 | . | 31.5431 |
| 14 |  | 1681.9 | 1.0000 | 7.16e-13 | 0.8750 | . | 15 | . | 34.3157 |
|  |  | Best | . | . | 0.8328 | . | 6 | 22.612 | 14.0201 |

^a^ SP, significant probability. ^b^ SSS, sequential sum of squares. ^c^ R^2^, coefficient of determination. ^e^ n, number of parameters in the model, including the intercept. ^f^ AICc, corrected akaike’s information criterion. ^g^ BIC, Bayesan information criterion.

**Table S11.** Stepwise variables result for multi-elemental analysis.

| **Step** |  | **Variables** | **SP^a^** | **SSS^b^** | **R^2c^** | **Cp^d^** | **n^e^** | **AICc^f^** | **BIC^g^** |
| --- | --- | --- | --- | --- | --- | --- | --- | --- | --- |
| 1 |  | S | 0.0000 | 4.511415 | 0.5827 | . | 2 | 24.7614 | 28.1745 |
| 2 |  | Ca | 0.0000 | 1.574923 | 0.7862 | . | 3 | 6.68807 | 10.8856 |
| 3 |  | Hg | 0.0004 | 0.630972 | 0.8677 | . | 4 | -5.3253 | -0.5553 |
| 4 |  | Mo | 0.0044 | 0.279373 | 0.9037 | . | 5 | -12.094 | -6.9905 |
| 5 |  | P | 0.0117 | 0.17017 | 0.9257 | . | 6 | -16.76 | -11.592 |
| 6 |  | Zr | 0.0005 | 0.231136 | 0.9556 | . | 7 | -29.019 | -24.093 |
| 7 |  | As | 0.0376 | 0.060089 | 0.9633 | . | 8 | -30.946 | -26.611 |
| 8 |  | Hf | 0.0337 | 0.053686 | 0.9703 | . | 9 | -33.016 | -29.676 |
| 9 |  | Be | 0.3423 | 0.009902 | 0.9715 | . | 10 | -29.485 | -27.606 |
| 10 |  | La | 0.0263 | 0.049239 | 0.9779 | . | 11 | -31.889 | -32.015 |
| 11 |  | U | 0.0185 | 0.044304 | 0.9836 | . | 12 | -35.105 | -37.875 |
| 12 |  | Al | 0.2379 | 0.009693 | 0.9849 | . | 13 | -30.734 | -36.908 |
| 13 |  | Ce | 0.2219 | 0.01011 | 0.9862 | . | 14 | -25.785 | -36.275 |
| 14 |  | Ba | 0.2950 | 0.0073 | 0.9871 | . | 15 | -19.12 | -35.033 |
| 15 |  | Mn | 0.1989 | 0.010707 | 0.9885 | . | 16 | -12.425 | -35.124 |
| 16 |  | Ni | 0.1763 | 0.011251 | 0.9900 | . | 17 | -4.6952 | -35.883 |
| 17 |  | Sm | 0.0698 | 0.017931 | 0.9923 | . | 18 | 1.25832 | -40.587 |
| 18 |  | Th | 0.2502 | 0.006479 | 0.9931 | . | 19 | 14.6082 | -40.712 |
| 19 |  | Pb | 0.3299 | 0.004597 | 0.9937 | . | 20 | 32.4764 | -40.076 |
| 20 |  | Cr | 0.3337 | 0.004551 | 0.9943 | . | 21 | 55.2654 | -39.687 |
| 21 |  | Si | 0.2711 | 0.005843 | 0.9951 | . | 22 | 84.074 | -40.659 |
| 22 |  | Lu | 0.1923 | 0.007737 | 0.9961 | . | 23 | 121.357 | -44.227 |
| 23 |  | Ti | 0.3297 | 0.004137 | 0.9966 | . | 24 | 178.843 | -45.307 |
| 24 |  | Sn | 0.2742 | 0.00513 | 0.9973 | . | 25 | 265.143 | -48.574 |
| 25 |  | Cu | 0.2570 | 0.005242 | 0.9979 | . | 26 | 411.366 | -53.917 |
| 26 |  | Rb | 0.3660 | 0.003299 | 0.9984 | . | 27 | 714.216 | -57.633 |
| 27 |  | Ga | 0.1975 | 0.006051 | 0.9991 | . | 28 | 1624.2 | -74.218 |
| 28 |  | Zn | 0.3886 | 0.002493 | 0.9995 | . | 29 | . | -85.296 |
| 29 |  | Bi | 0.4865 | 0.002176 | 0.9997 | . | 30 | . | -104.69 |
|  |  | Best | . | . | 0.9997 | . | 30 | . | -104.69 |

^a^ SP, significant probability. ^b^ SSS, sequential sum of squares. ^c^ R^2^, coefficient of determination. ^e^ n, number of parameters in the model, including the intercept. ^f^ AICc, corrected akaike’s information criterion. ^g^ BIC, Bayesan information criterion.

**References**

1. Cocozza, C., D'orazio, V., Miano, T.M. & Shotyk, W. Characterization of solid and aqueous phases of a peat bog profile using molecular fluorescence spectroscopy, ESR and FT-IR, and comparison with physical properties. Org. Geochem. 34(1), 49-60 (2003).
2. Kloprogge, J.T., Hickey, L. & Frost, R.L. FT‐Raman and FT‐IR spectroscopic study of synthetic Mg/Zn/Al‐hydrotalcites. J. Raman Spectros. 35(11), 967-974 (2004).
3. Niemeyer, J., Chen, Y., & Bollag, J.M. Characterization of humic acids, composts, and peat by diffuse reflectance Fourier‐transform infrared spectroscopy. Soil Sci. Soc. Am. J. 56(1), 135-140 (1992).
4. Romão, L.P. et al. Structure and properties of Brazilian peat: analysis by spectroscopy and microscopy. J. Braz. Chem. Soc. 18, 714-720 (2007).
5. Haberhauer, G., Rafferty, B., Strebl, F. & Gerzabek, M.H. Comparison of the composition of forest soil litter derived from three different sites at various decompositional stages using FTIR spectroscopy. Geoderma 83(3-4), 331-342 (1998).
6. Gondar, D., Lopez, R., Fiol, S., Antelo, J.M. & Arce, F. Characterization and acid–base properties of fulvic and humic acids isolated from two horizons of an ombrotrophic peat bog. Geoderma 126, 367–374 (2005).
7. Ibarra, J.V., Munoz, E. & Moliner, R. FTIR study of the evolution of coal structure during the coalification process. Org. Geochem. 24, 725–735 (1996).
8. Zaccheo, P., Cabassi, G., Ricca, G. & Crippa, L. Decomposition of organic residues in soil: experimental technique and spectroscopic approach. Org. Geochem. 33, 327–345 (2002).
9. Coates, J.P. The interpretation of infrared spectra: Published reference sources. Appl. Spectros. Rev. 31(1-2), 179-192 (1996).
10. Parker, F. Applications of infrared spectroscopy in biochemistry, biology, and medicine (Springer Science & Business Media, 2012).
11. Guo, Y. & Bustin, R.M. FTIR spectroscopy and reflectance of modern charcoals and fungal decayed woods: implications for studies of inertinite in coals. Int. J. Coal Geol. 37(1-2), 29-53 (1998).
12. González, J.A. et al. Preferential accumulation of selectively preserved biomacromolecules in the humus fractions from a peat deposit as seen by analytical pyrolysis and spectroscopic techniques. J. Anal. Appl. Pyrolysis 68, 287-298 (2003).
13. Smith, B. Infrared spectral interpretation: a systematic approach (CRC press, 2018).
14. Zaccone, C., Miano, T.M. & Shotyk, W. Qualitative comparison between raw peat and related humic acids in an ombrotrophic bog profile. Org. Geochem. 38(1), 151-160 (2007).
15. Rossel, R.V. & Behrens, T. Using data mining to model and interpret soil diffuse reflectance spectra. Geoderma 158(1-2), 46-54 (2010).
16. Hafidi, M., Amir, S., Revel, J.C. Structural characterization of olive mill waster-water after aerobic digestion using elemental analysis, FTIR and 13C NMR. Process Biochem. 40(8), 2615-2622 (2005).
17. Poppi, N.R. & Talamoni, J. Estudo dos ácidos húmico e fúlvico, extraídos de solos, por espectroscopia de infravermelho. Quím. Nova 15(4), 281-285 (1992).
18. Marchessault, R.H. Application of infra-red spectroscopy to cellulose and wood polysaccharides. Pure Appl. Chem. 5(1-2), 107-130 (1962).
19. Hergert, L. in: Infrared spectra, Lignins (eds. Sarkanen, K., Ludwin, C.). (Wiley Interscience, 1974).
20. Painter, P., Starsinic, M. & Coleman, M. Determination of functional groups in coal by Fourier transform interferometry. Fourier Transform Infrared Spectros. 4, 169-240 (2012).
21. Grube, M., Lin, J.G., Lee, P.H. & Kokorevicha, S. Evaluation of sewage sludge-based compost by FT-IR spectroscopy. Geoderma 130(3-4), 324-333 (2006).
22. Geiger, C.A. A powder infrared spectroscopic investigation of garnet binaries in the system Mg3Al2Si3O12-Fe3Al2O12-Mn3Al2Si3O12-Ca3Al2Si3O12 (1998).
23. Saikia, B.J., Parthasarathy, G. & Sarmah, N.C. Fourier transform infrared spectroscopic estimation of crystallinity in SiO2 based rocks. Bull. Mater. Sci. 31(5), 775-779 (2008).
24. Orlov, D.S. Humus Acids of Soils, Russian Translation Series 35. A. A. Balkema: Rotterdam, The Netherlands, 35-70 (1985).

25. Haberhauer, G., Feigl, B., Gerzabek, M.H. & Cerri, C. FT-IR spectroscopy of organic matter in tropical soils: changes induced through deforestation. Appl. Spectros. 54(2), 221-224 (2000).
